# Targeting HuR-Vav3 mRNA interaction prevents *Pseudomonas aeruginosa* adhesion to the cystic fibrosis airway epithelium

**DOI:** 10.1172/jci.insight.161961

**Published:** 2023-02-08

**Authors:** Mehdi Badaoui, Cyril Sobolewski, Alexandre Luscher, Marc Bacchetta, Thilo Köhler, Christian van Delden, Michelangelo Foti, Marc Chanson

**Affiliations:** 1 Department of Cell Physiology & Metabolism and; 2 Department of Microbiology & Molecular Medicine, Faculty of Medicine, University of Geneva, Switzerland.

**Keywords:** Cell Biology, Infectious disease, Extracellular matrix, Fibronectin, Transcription

## Abstract

Cystic fibrosis (CF) is characterized by chronic bacterial infections leading to progressive bronchiectasis and respiratory failure. *Pseudomonas aeruginosa* (*Pa*) is the predominant opportunistic pathogen infecting the CF airways. The guanine nucleotide exchange factor Vav3 plays a critical role in *Pa* adhesion to the CF airways by inducing luminal fibronectin deposition that favors bacteria trapping. Here we report that Vav3 overexpression in CF is caused by upregulation of the mRNA-stabilizing protein HuR. We found that HuR accumulates in the cytoplasm of CF airway epithelial cells and that it binds to and stabilizes Vav3 mRNA. Interestingly, disruption of the HuR-Vav3 mRNA interaction improved the CF epithelial integrity, inhibited the formation of the fibronectin-made bacterial docking platforms, and prevented *Pa* adhesion to the CF airway epithelium. These findings indicate that targeting HuR represents a promising antiadhesive approach in CF that can prevent initial stages of *Pa* infection in a context of emergence of multidrug-resistant pathogens.

## Graphical abstract

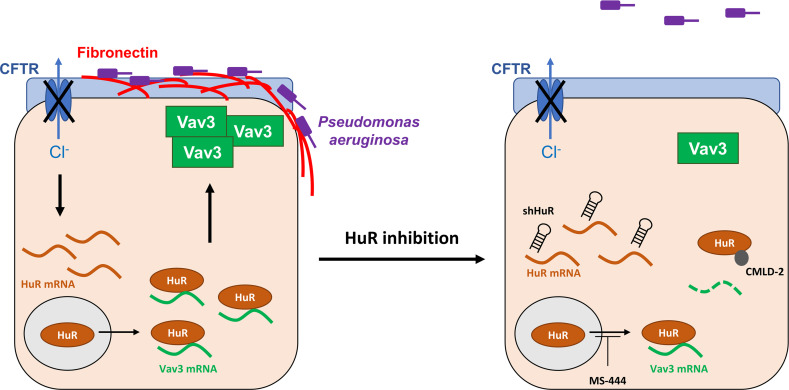

## Introduction

Cystic fibrosis (CF) is a genetic disease caused by mutations of the gene coding for the CS transmembrane conductance regulator (CFTR) anion channel. CF affects more than 70,000 people worldwide and leads to severe respiratory and digestive disorders (1). Most often, infections of the respiratory tract are prominent and determine the severity of the disease. Chronic lung infection by the opportunistic pathogen *Pseudomonas aeruginosa* (*Pa*) represents the first cause of morbidity and mortality in CF. Recurrent *Pa* infections are associated with pulmonary exacerbations, hospital admissions, and lung function decline, with lung transplantation being, at present, the only alternative to improve median survival of patients having advanced CF disease. However, the mechanisms responsible for this predisposition to *Pa* infection are not fully understood. Among other hypotheses, airway mucus obstruction by the airway surface liquid dehydration and neutrophil extracellular traps (2), impaired mucociliary clearance (3), and decreased bacteria killing by lack of sphingosine (4) or antimicrobial peptide inactivation have been described (5). We recently reported that the guanine nucleotide exchange factor (GEF) Vav3, which belongs to the family of Rho GTPase activators, is upregulated in CF. Vav3 overexpression mediates the formation of luminal “bacterial docking stations” rich in fibronectin, and these “docking stations” promote the adhesion of *Pa* to the CF airway epithelium surface (6). Importantly, Vav3 silencing in CF human airway epithelial cells (HAECs) inhibited fibronectin surface expression and prevented *Pa* adhesion to the epithelium.

Fibronectin, a component of the extracellular matrix, is a known target for pathogens providing a substrate for bacterial adhesion and trapping in proximity to the epithelium surface (7, 8). The transition from a nonattached to an attached state promotes irreversible anchoring of *Pa* to the airway surface (9). This step offers protection to the bacteria against antibiotics and other bactericidal molecules that makes these infections difficult to eradicate (10). Due to repeated antibiotic treatment courses in *Pa*-infected individuals, multidrug-resistant isolates are frequently encountered. Treatment options are limited due to cross-resistance mechanisms and to the limited availability of novel antimicrobial molecules. Hence, alternative treatment strategies are urgently needed. Deciphering the molecular mechanism linking CFTR dysfunction to Vav3 overexpression and the subsequent formation of fibronectin adhesion platforms will pave the way for alternative approaches with which to tackle the infection-prone phenotype of CF lungs.

Vav3 is a signal transducer in actin organization that constitutes an established marker for several cancers (11). While its upregulation is described in different tumors (12–17), the precise mechanism underlying Vav3 overexpression has not yet been determined. This altered expression can be regulated at both transcriptional and posttranscriptional levels. It has been proposed that Vav3 overexpression in gastric cancer is mediated by increased mRNA stability (18). For instance, posttranscriptional gene regulation process is highly regulated by RNA binding proteins (RBPs) (19), which control mRNA localization, stability, and degradation (20). Among the RBPs, human antigen R (HuR) and tristetrapolin (TTP) are the most studied. Both proteins competitively bind to the 3′UTR of mRNAs expressing the specific sequences enriched in adenosine and uridine (AU-rich elements [AREs]) (21). When activated, HuR shuttles from the nucleus to the cytoplasm and promotes mRNA stability and translation (22), while TTP activation promotes mRNA decay (23). The aberrant expression and localization of HuR/TTP is linked to the altered expression of genes containing AREs and the development of various diseases.

Several reports highlighted the involvement of RBPs in the development and progression of cancers, inflammatory diseases, and fibrosis (24–27). There is growing evidence that posttranscriptional gene regulation contributes to the CF nongenomic instability. While many studies focused on the role of miRNAs and lncRNAs on CFTR-regulated genes (28), the function of RBPs in CF is still unclear. Here, we show that HuR plays an essential role in the adhesion of *Pa* to the CF airway surface by controlling Vav3 posttranscriptional regulation. CFTR mutation or knockdown correlated with HuR upregulation and cytoplasmic accumulation. By combining bioinformatic analysis and ribonucleoprotein immunoprecipitation (RNP-IP), we demonstrate that HuR interacted physically with Vav3 mRNA. This interaction was increased in CF cells, promoting Vav3 mRNA stability. Blocking HuR-Vav3 mRNA interaction prevented Vav3 overexpression, fibronectin apical deposition, and consequently, *Pa* adhesion to the CF epithelium. Our results anticipate potentially new therapeutic approaches targeting the initial and decisive phase of *Pa* colonization, which is crucial to the improvement of later lung function and the quality of life of people with CF.

## Results

### HuR is overexpressed in primary CF HAECs.

**Figure 1:**
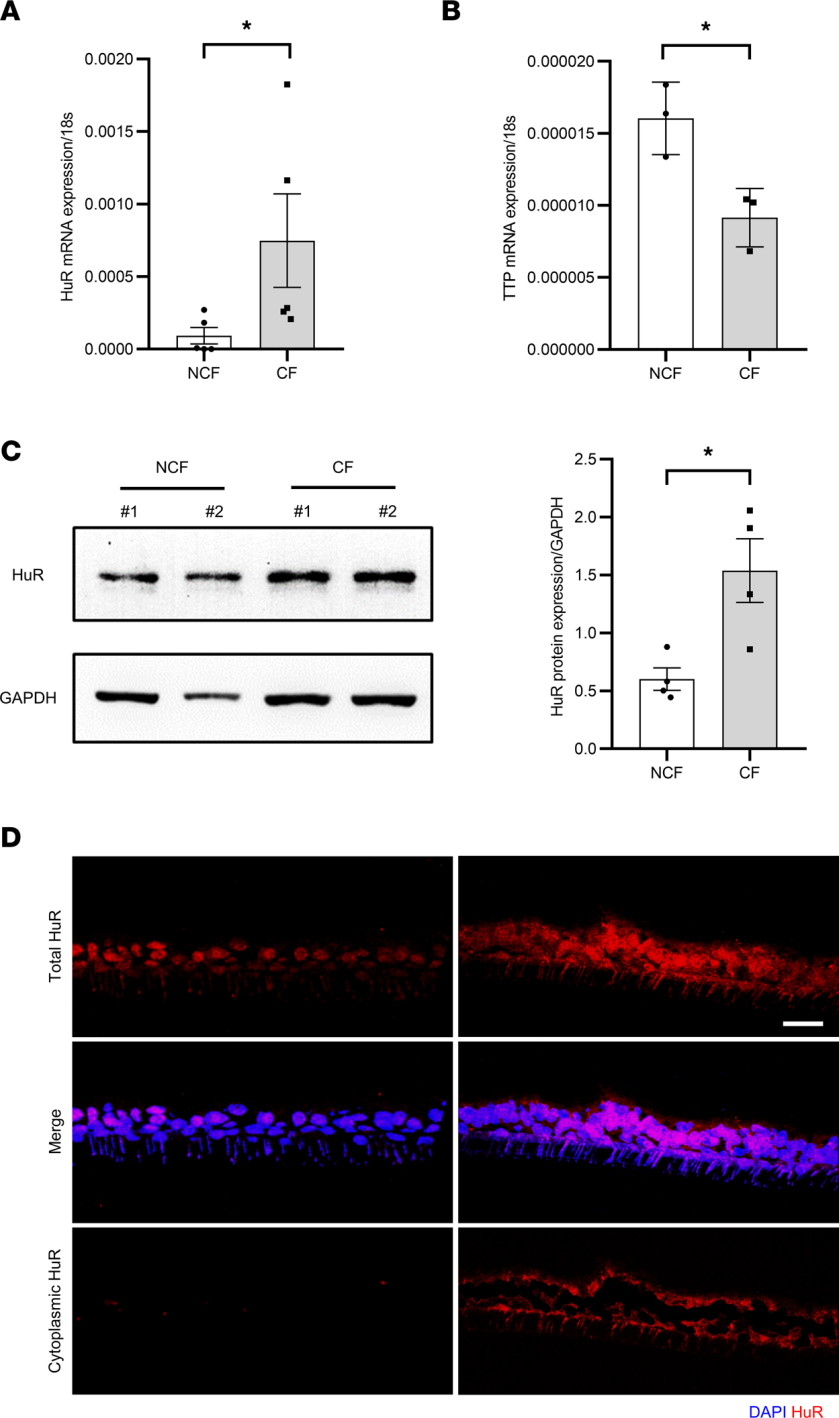
Increased HuR expression and cytoplasmic accumulation in CF. (**A** and **B**) Analysis of the relative mRNA expression of HuR (**A**) and TTP (**B**) by qPCR in CF versus NCF fully differentiated primary HAECs. 18s served as an internal control. *N* = 5 donors in each group for HuR, and *N* = 3 donors in each group for TTP. Mann-Whitney *U* test, *P < 0.05. (**C**) Representative Western blot showing HuR expression in CF versus NCF fully differentiated primary HAECs. GAPDH served as an internal control. Representative blots are shown on the left panels, and the quantification is shown on the right panel. *N* = 4 donors in each group. Unpaired 2-tailed Student’s *t* test, *P < 0.05. (**D**) Representative confocal images of HuR immunostaining (red) on cryosections of CF and NCF fully differentiated primary HAECs. Nuclei are stained in blue. The cytoplasmic staining of HuR was obtained by subtracting the nuclear signal using the volume mask function of IMARIS software. Scale bar: 30 μm.

The RNA binding proteins HuR and TTP exhibit competitive binding capacities and opposite functions. While HuR stabilizes ARE-containing mRNA, TTP promotes their degradation. To evaluate their potential involvement in Vav3 overexpression in CF, we analyzed their expression on primary HAECs isolated from CF donors homozygous for the F508del-CFTR mutation. These cells were grown on Transwell filters and differentiated at air-liquid interface (ALI). As shown in Figure 1A, HuR mRNA expression was significantly increased in CF cells (Figure 1A). In contrast, TTP mRNA and protein expression was decreased in CF (Figure 1B and Supplemental Figure 1A; supplemental material available online with this article; https://doi.org/10.1172/jci.insight.161961DS1). HuR overexpression was confirmed at the protein level in CF primary HAECs by Western blot (Figure 1C) and immunostaining (Figure 1D). In addition to its overexpression, confocal microscopy revealed a distinct localization of HuR in CF. While a clear nuclear localization was detected in non-CF (NCF) primary cultures, we observed a strong redistribution of HuR in the cytoplasm of CF HAECs. These results indicate that HuR is overexpressed in F508del-CFTR primary HAECs, with an abnormal accumulation in the cytoplasm.

### CFTR silencing induces HuR overexpression.

**Figure 2:**
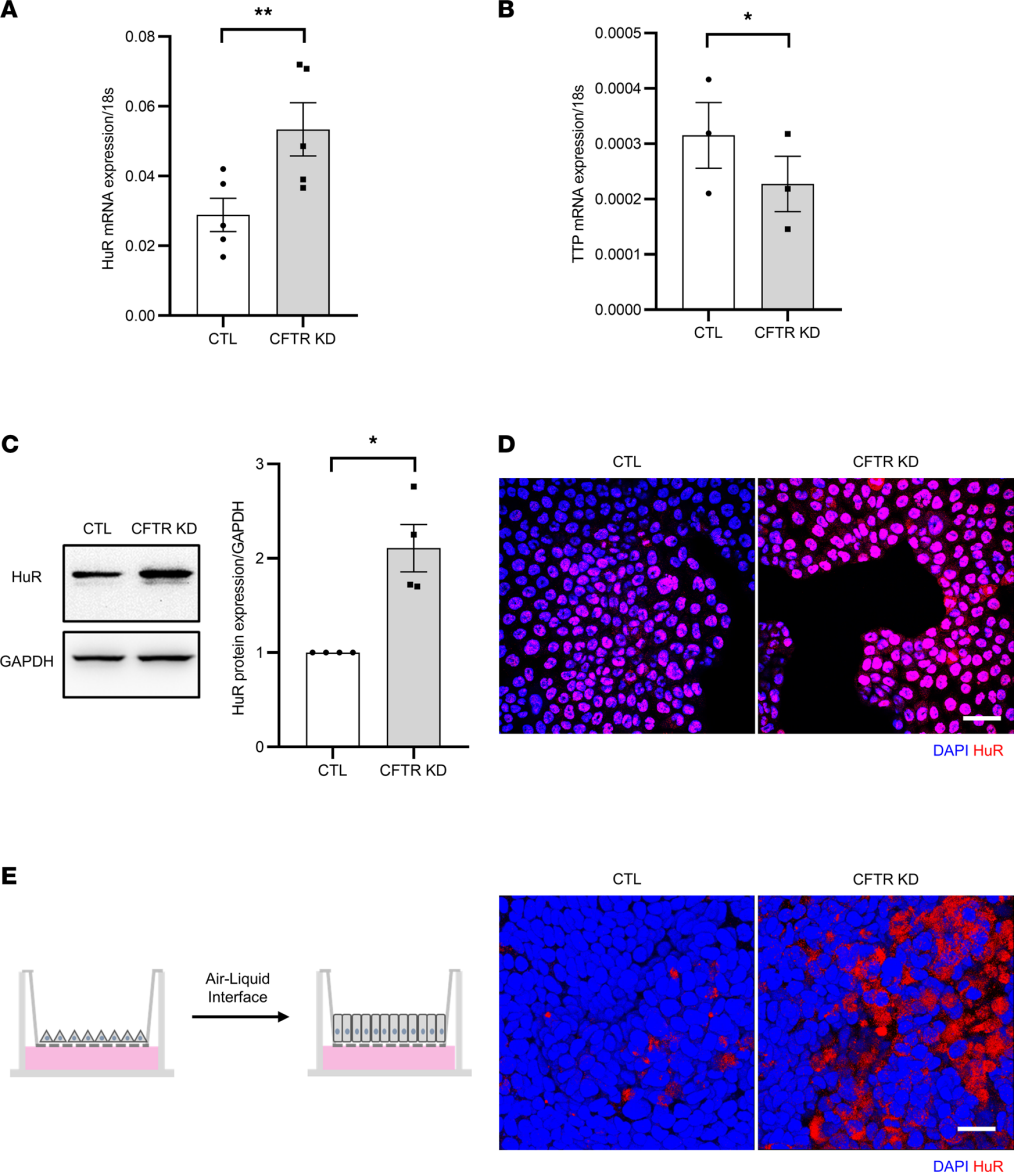
CFTR knockdown in Calu-3 cells induces HuR overexpression. (**A** and **B**) Quantification of the relative mRNA expression of HuR (**A**) and TTP (**B**) by qPCR in CFTR KD cells versus CTL Calu-3 cells. 18s served as an internal control. *n* = 5 in each group for HuR, and *n* = 3 in each group for TTP. Unpaired 2-tailed Student’s *t* test, *P < 0.05. (**C**) Representative Western blot showing HuR expression in CFTR KD cells versus CTL Calu-3 cells. GAPDH served as an internal control. Representative blots are shown on the left panels, and the quantification is shown on the right panel. *n* = 4 in each group. Mann-Whitney *U* test, *P < 0.05. (**D**) Representative confocal images of HuR (red) immunostaining in monolayer cultures of CFTR KD cells versus CTL Calu-3 cells. Nuclei are shown in blue. (**E**) Confocal microscopy analysis of HuR (red) localization in polarized CFTR KD cells versus CTL Calu-3 cells at ALI. Right panels show top view of representative images from 3D reconstruction of *Z* stack data. Scale bars: 20 μm.

To assess whether there is a causal relationship between CFTR dysfunction and HuR overexpression, we determined HuR expression after *CFTR* knockdown by CRISPR-Cas9 gene editing (CFTR KD) in the Calu-3 airway epithelial cell line (29). Similarly to the primary CF HAECs, an increased HuR expression (Figure 2A) at the mRNA level was observed in CFTR KD cells grown as a monolayer in comparison with control cells (CTL cells). In addition, Western blot (Figure 2C) and confocal microscopy analysis (Figure 2D) confirmed HuR overexpression in the CFTR KD cells at the protein level. TTP was, however, decreased in terms of mRNA (Figure 2B) and protein (Supplemental Figure 1B) expression in CFTR KD cells. HuR overexpression with enriched redistribution in the cytoplasm was maintained in polarized CFTR KD cells grown at ALI (Figure 2E). To support the microscopic observation of a different HuR compartmentalization in CFTR KD cells, protein extracts were fractionated into cytoplasmic and nuclear fractions before (monolayers) and after polarization at ALI. As shown in Supplemental Figure 2A, the purity of the cytosolic and the nuclear fractions was verified by Western blot against GAPDH and Histone H3, respectively (left panels, *n* = 3). Again, in addition to the overexpression, we found an increased HuR amount in the cytosolic fraction of CFTR KD, as compared with CTL cells (right panels, *n* = 3).

Different mutation classes are reported in CF affecting CFTR expression, processing, or gating. Interestingly, HuR expression was also increased in HeLa cells expressing G551D-CFTR, which is the most common CFTR gating mutation, as compared with cells expressing WT-CFTR (Supplemental Figure 2B). These results suggest that HuR overexpression and cytoplasmic localization is directly caused by CFTR dysfunction.

### HuR binds to Vav3 mRNA and promotes its stability in CFTR KD cells.

**Figure 3:**
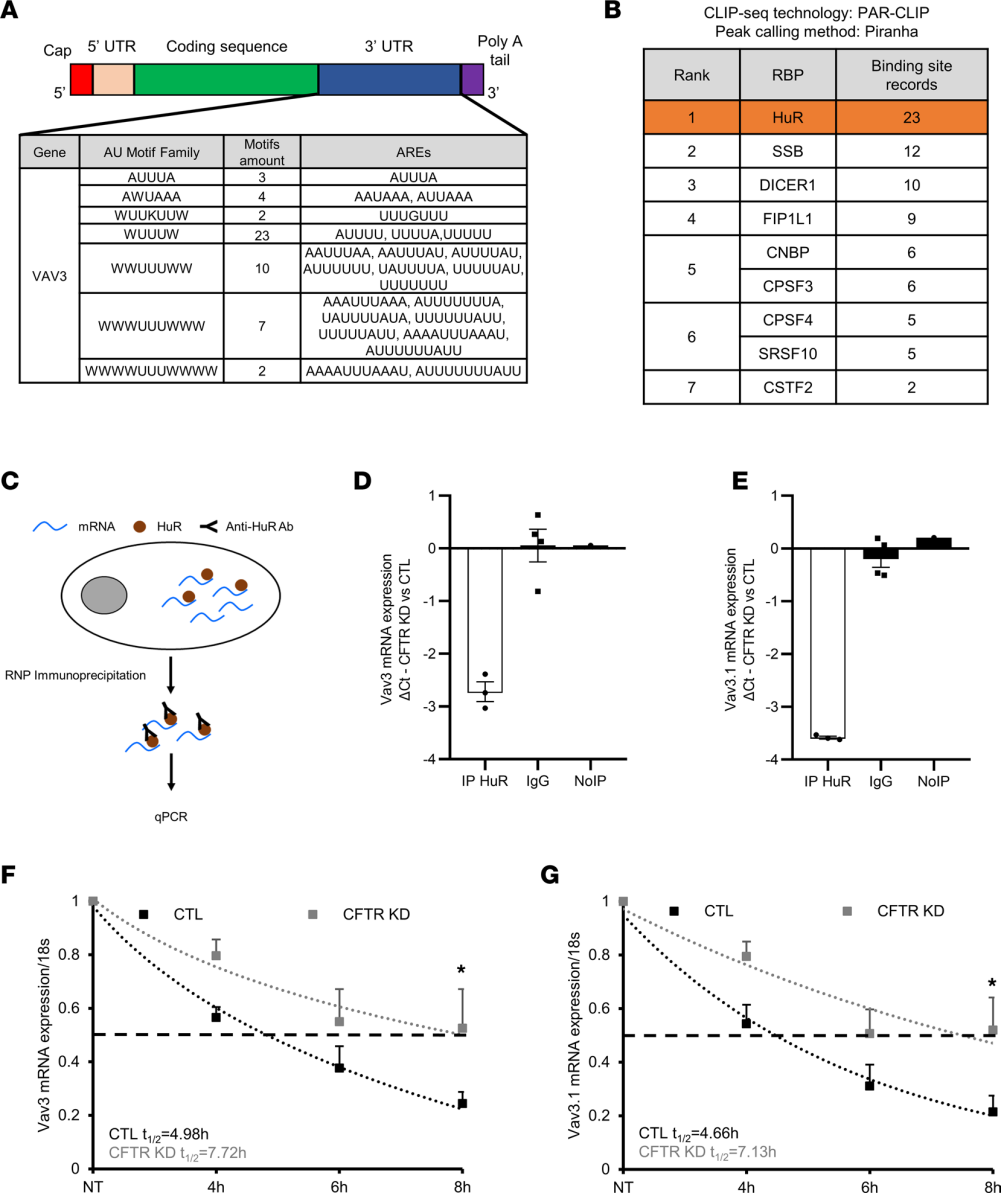
Enriched interaction between HuR and Vav3 transcripts in CFTR KD Calu-3 cells promotes Vav3 mRNA stability. (**A**) Data showing the different AREs motifs expressed by Vav3 at the 3′UTR region. (**B**) The number of the predicted binding sites for RBPs recorded on Vav3 mRNA 3′UTR region. HuR, which ranked no. 1, is highlighted in orange. (**C**) Schematic representation of the protocol used to immunoprecipitate the mRNA bound to HuR. (**D** and **E**) Quantification of Vav3 (**D**) and its isoform Vav3.1 (**E**) expression in the pool of mRNA bound to HuR in CFTR KD cells versus CTL Calu-3 cells by qPCR after HuR RNP. Values are represented as mean ΔCt (Ct_CFTR_ _KD_ – Ct_CTL_) of 3 independent replicates. The IgG isotype control antibody or the omission of the immunoprecipitant antibody (NoIP) conditions served as negative controls. (**F** and **G**) Vav3 and Vav3.1 mRNA decay was analyzed by qPCR in CFTR KD versus CTL Calu-3 cells after inhibition of the de novo transcription by actinomycin-D. *n* = 3 in each group. Unpaired 2-tailed Student’s *t* test, *P < 0.05.

AREs in the 3′UTRs are important regions for posttranscriptional gene regulation through their capacity to form specific binding sites for RBPs, including HuR and TTP (30). To identify a potential interaction between HuR and Vav3 mRNA, we first verified whether the Vav3 mRNA sequence contains AREs. The analysis of the 3′UTR region of Vav3 mRNA by the ARE site database (31) revealed the presence of 51 AREs represented by different classes of AU motifs (Figure 3A). Indeed, the pentamer AUUUA is the core of the AREs, but this motif is not functional alone and requires regions surrounded by AU motifs like heptamers (WWUUUWW), nanomers (WWWUUUWWW), and undecamers (WWWWUUUWWWW) (32). Next, we searched for the different binding sites for RBPs expressed by Vav3 mRNA at the 3′UTR region. To this end, the predicted binding sites were identified by cross-linking immunoprecipitation high-throughput next-generation sequencing (CLIP-Seq) experiments from multiple large-scale high-throughput sequencing data sets integrated in the POSTAR3 database (33). Interestingly, we detected the presence of several binding sites for several RBPs, including HuR (Figure 3B). Importantly, HuR represents the top candidate, since 23 binding sites for HuR were recorded on Vav3 mRNA at the 3′UTR region, whereas the bioinformatic analysis did not predict TTP as a potential RBP for Vav3 mRNA.

To confirm the interaction between HuR and Vav3 mRNA, we performed RNP-IP to isolate the mRNA specifically bound to HuR in CTL and CFTR KD Calu-3 cells (Figure 3C). Western blots of the whole cell lysate and after HuR IP confirmed the increased expression of HuR in CFTR KD cells (Supplemental Figure 3A). As shown in Supplemental Figure 3A, no HuR was immunoprecipitated with the negative control condition where the IP antibody was omitted (NoIP). Next, total mRNA extraction after HuR pull-down was performed and Vav3 mRNA expression verified by quantitative PCR (qPCR). To avoid any bias due to a potential unspecific interaction between the beads, the antibody, and mRNAs, a preclearing step of the beads was performed in addition to the use of an anti–flag IgG rabbit antibody. Thus, no Vav3 mRNA enrichment was observed in the IgG or the NoIP negative control (Figure 3D). In contrast, we detected Vav3 mRNA in the RNP complex, confirming its interaction with HuR. The amount of Vav3 mRNA was strongly increased by 2.74 Ct in the CFTR KD cells in comparison with CTL cells, suggesting that the interaction between HuR and Vav3 mRNA is enriched in CF (Figure 3D). There are 2 Vav3 isoforms — the full length Vav3 and the N-terminal truncated version Vav3.1 — obtained by alternative splicing and without known function (34). To assess the specificity of our IP assay, we used primers targeting Vav3.1 to verify that the amount of Vav3.1 mRNA interacting with HuR was also increased (3.58 Ct in CFTR KD cells when compared with CTL cells; Figure 3E). To evaluate the consequence of enriched HuR interaction with Vav3 mRNA in CFTR KD cells, we measured Vav3 mRNA decay by actinomycin-D chase experiments. Interestingly, Vav3 mRNA stability was significantly increased in the CFTR KD cells 8 hours after actinomycin-D treatment (Figure 3F). Similar results were obtained regarding Vav3.1 mRNA stability (Figure 3G). Indeed, the half-life of Vav3 and Vav3.1 was respectively increased by 2.74 hours and 2.47 hours in the CFTR KD when compared with CTL cells.

As an attempt to determine if more HuR was bound to each Vav3 mRNA or if there was stronger HuR binding to Vav3 mRNA, we subjected the recovered mRNA to high-throughput next-generation sequencing (RNA-Seq). In addition to the NoIP condition, CTL and CFTR KD cells were treated with CMLD-2 (35), an inhibitor that competitively binds to HuR and disrupts its interaction with AREs (Supplemental Figure 3B). Next, we mapped the 3′UTR region of Vav3 mRNA and determined the number of reads at potential sites of interaction with HuR. Quantification revealed 748 reads detected in the CTL cells, while 1,189 reads were sequenced in the CFTR KD cells after HuR pull-down, confirming the enriched interaction between HuR and Vav3 mRNA by 1.58-fold. As shown in Supplemental Figure 3C, we found that the same 3′UTR Vav3 mRNA regions were mapped in both CTL and CFTR KD cells, with higher signal in CFTR KD cells (red boxes). In addition, potentially novel peaks of HuR-Vav3 mRNA interaction were detected in other 3′UTR regions only in the CFTR KD conditions when compared with CTL (black boxes; Supplemental Figure 3C). Importantly theses mapped regions were not detected in the presence of CMLD-2 and in the NoIP conditions. These results suggest that HuR overexpression in CF cells increases Vav3 mRNA stability through its binding to the 3′UTR AREs.

Both TTP and HuR can competitively bind to AREs (36). To determine whether TTP contributes to Vav3 posttranscriptional regulation, we overexpressed TTP in Calu-3 cells by means of a lentiviral vector. Stable TTP overexpression (TTP^OE^) in CTL and CFTR KD cells was confirmed at the protein level by Western blot without affecting HuR expression (Supplemental Figure 4A). In addition, TTP^OE^ had no effect on Vav3 and Vav3.1 transcript levels in CTL and CFTR KD, suggesting that TTP^OE^ did not restore Vav3 posttranscriptional imbalance (Supplemental Figure 4B). Finally, the main proteins regulated by Vav3 in CF, namely fibronectin and β1 integrin (Supplemental Figure 4A), or the transepithelial electrical resistance (TEER) — an indicator of epithelial integrity of polarized Calu-3 (Supplemental Figure 4C) — were not altered by TTP^OE^. These observations suggest that Vav3 posttranscriptional regulation in Calu-3 cells is independent from TTP.

### HuR inhibition prevented Vav3 overexpression in CFTR KD cells.

**Figure 4:**
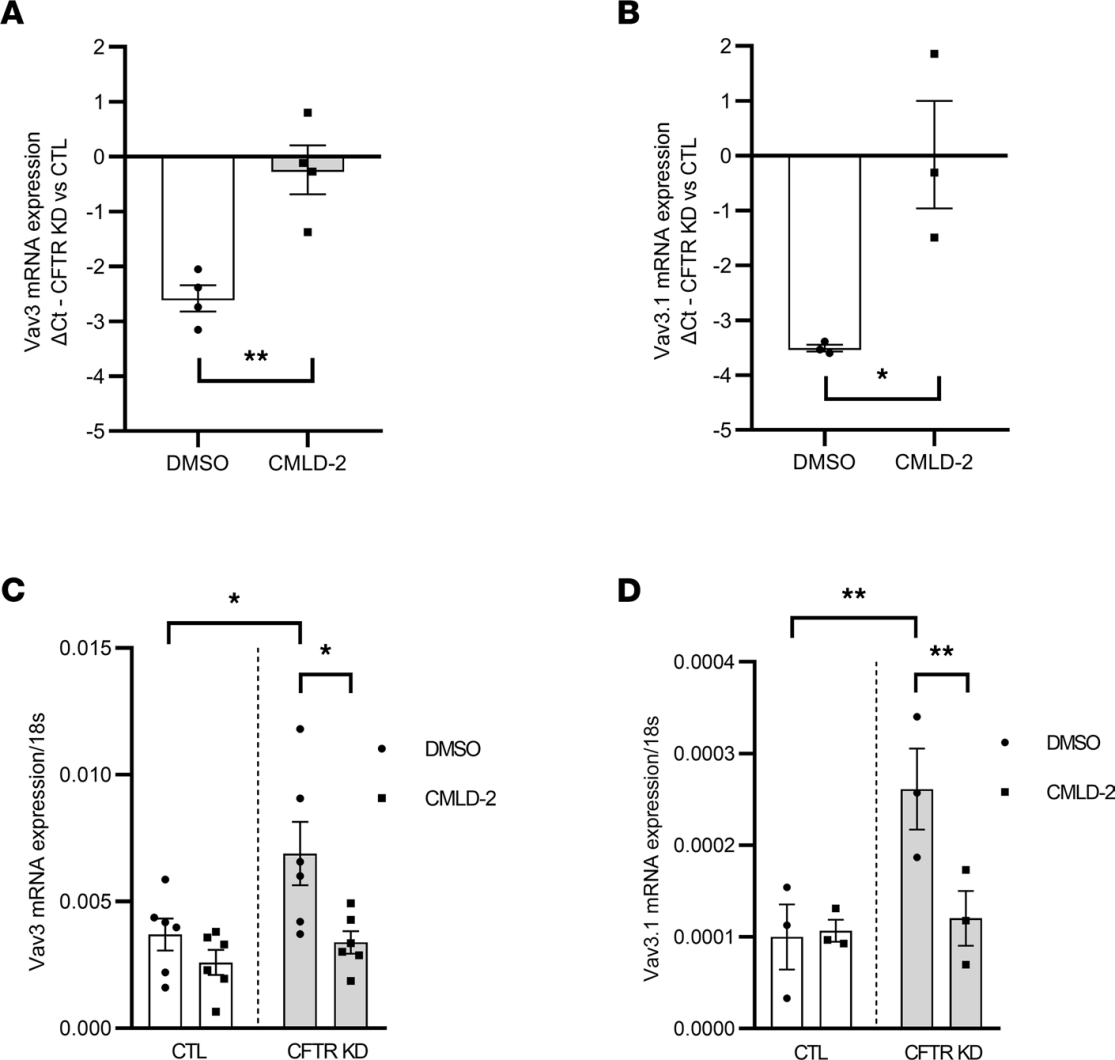
Disrupting HuR interaction with Vav3 transcripts by CMLD-2 inhibits Vav3 mRNA upregulation in CFTR KD Calu-3 cells. (**A** and **B**) Quantification of Vav3 (**A**) and Vav3.1 (**B**) interaction with HuR by qPCR after HuR RNP in CFTR KD cells versus CTL Calu-3 cells after CMLD-2 treatment. *n* = 4 in each group for Vav3 and *n* = 3 in each group for Vav3.1. Unpaired 2-tailed Student’s *t* test, *P < 0.05, **P < 0.01. (**C** and **D**) Quantification of the relative mRNA expression of Vav3 (**C**) and Vav3.1 (**D**) by qPCR in CFTR KD cells versus CTL Calu-3 cells treated with CMLD-2. 18s served as an internal control. *n* = 5 in each group for Vav3, and *n* = 3 in each group for Vav3.1. Two-way ANOVA, *P < 0.05, **P < 0.01.

To investigate the potential role of HuR on Vav3 overexpression in CF, CTL and CFTR KD cells were treated with CMLD-2 at 20 μM or 35 μM to determine whether these concentrations were able to prevent HuR interaction with Vav3 mRNA after 24 hours. In agreement with the Vav3 mRNA 3′UTR mapping data (Supplemental Figure 3C), qPCR experiments confirmed that CMLD-2 treatment significantly inhibited the enriched interaction between Vav3 mRNA and HuR (Figure 4A), whatever the concentration. Again, a similar effect was found on Vav3.1 interaction with HuR (Figure 4B). It has been reported that CMLD-2 could display selective cytotoxicity in some cell lines (37). To address this possibility, we measured LDH activity upon CMLD-2 treatment of Calu-3 cells. First, we determined the optimum cell number for the LDH assay (Supplemental Figure 5A). Second, we measured the maximum and the spontaneous LDH activity. As shown in Supplemental Figure 5, B and C, CMLD-2 used at 20 μM or 35 μM did not display any cytotoxicity against CTL and CFTR KD cells 24 hours after CMLD-2 treatment. Since CMLD-2 was able to prevent HuR interaction with Vav3 and Vav3.1 mRNAs, we next investigated the effect of HuR inhibition on Vav3 and Vav3.1 overexpression in CF. To this end, qPCR experiments were performed on CTL and CFTR KD cells treated or not with CMLD-2 for 24 hours. Interestingly, Vav3 and Vav3.1 mRNAs were significantly increased in the nontreated CFTR KD cells when compared with their respective CTL cells. However, blocking HuR-ARE interactions reduced Vav3 and Vav3.1 mRNA expression to levels observed in CTL cells, suggesting that HuR overexpression in CF is responsible of the increased Vav3 mRNA stability in CFTR KD cells (Figure 4, C and D).

It has been reported that HuR can regulate the expression of proinflammatory genes (38). To evaluate the consequence of HuR overexpression on the immune function of the airway epithelium, we evaluated the mRNA stability of *IL-6* and *CXCL-8* (*IL-8*), 2 major cytokines involved in the characteristic exacerbated inflammation in CF (39). Actinomycin-D chase experiments revealed that IL-6 mRNA decay was not altered in the CFTR KD cells (Supplemental Figure 6A) while IL-8 mRNA stability was significantly increased at 4 hours after actinomycin-D treatment (Supplemental Figure 6B). However, the latter effect was transient, and no significant difference was observed at 8 hours after actinomycin-D treatment. In addition, we measured IL-6 and IL-8 mRNA expression by qPCR in CFTR KD and CTL cells treated with CMLD-2 for 24 hours. The amounts of IL-6 and IL-8 transcripts were not affected by CMLD-2 treatment (Supplemental Figure 6, C and D). These findings suggest that HuR-induced mRNA stability displays selectivity regarding Vav3 mRNA decay regulation without affecting IL-6 and IL-8 transcripts.

### HuR inhibition restored the epithelial integrity and inhibited fibronectin ectopic deposition in CFTR KD cells.

**Figure 5:**
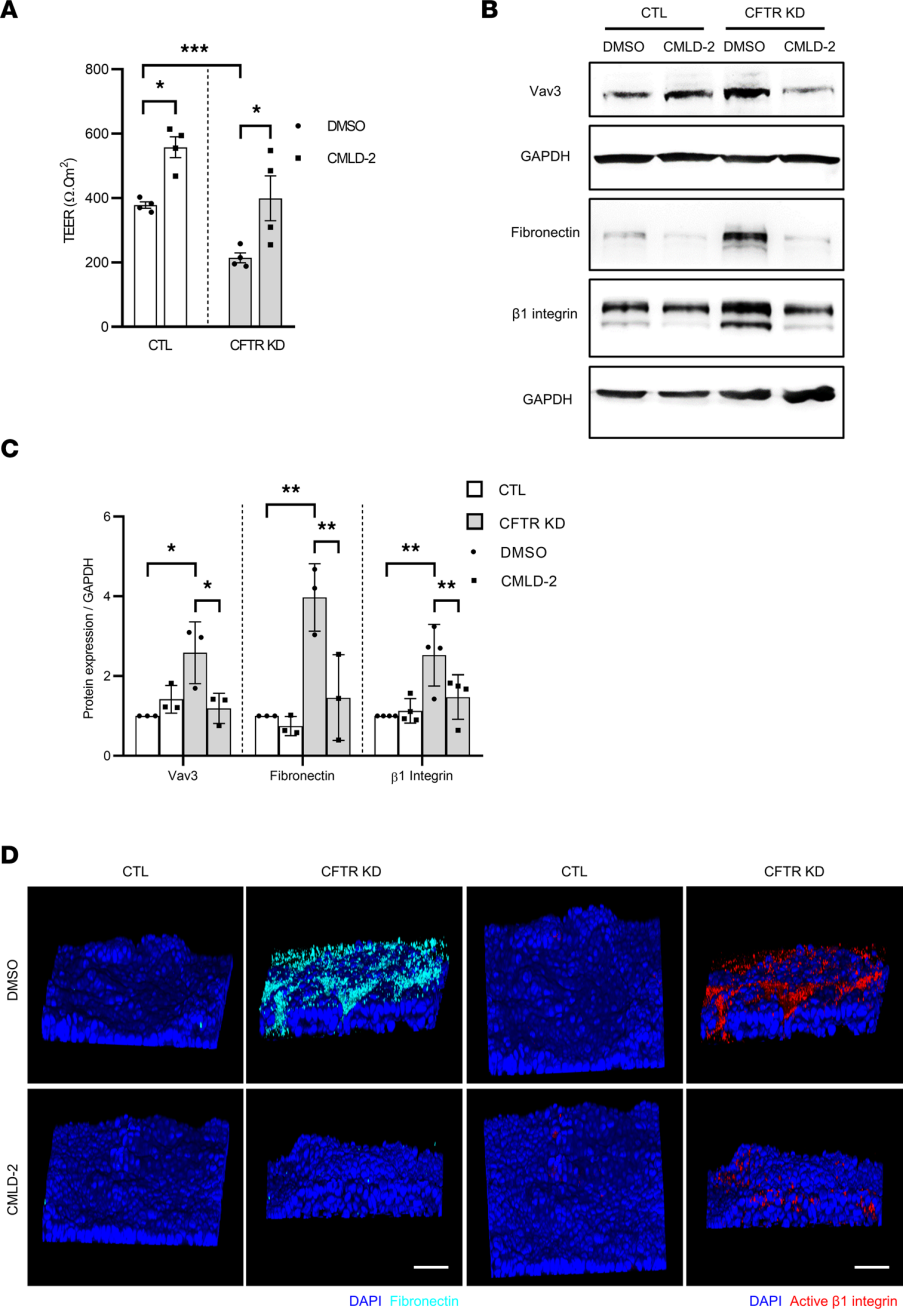
CMLD-2 treatment inhibited fibronectin deposition and improved the integrity of CFTR KD epithelium. (**A**) Transepithelial electrical resistance (TEER) measurements in polarized CFTR KD cells versus CTL Calu-3 cells at ALI-treated with CMLD-2. *n* = 4 in each group. Two-way ANOVA. *P < 0.05, ***P < 0.001. (**B** and **C**) Representative Western blot (**B**) and the corresponding quantifications (**C**) showing Vav3, fibronectin, and β1 integrin expression in CFTR KD cells versus CTL Calu-3 cells treated with CMLD-2. GAPDH served as an internal control. *n* = 3 in each group for Vav3, and *n* = 3 in each group for fibronectin and β1 integrin. Two-way ANOVA, *P < 0.05, **P < 0.01. (**D**) Confocal microscopy analysis of fibronectin (cyan) and active β1 integrin (red) localization in polarized CFTR KD cells versus CTL Calu-3 cells at ALI. Right panels show representative images from 3D reconstruction of *Z* stack data. Scale bars: 50 μM.

We have previously reported that Vav3 overexpression alters the CF epithelial integrity through its capacity to regulate cytoskeleton remodeling (6). Therefore, we tested whether preventing Vav3 overexpression in CFTR KD by targeting HuR would improve epithelial integrity of Calu-3 cells. First, CFTR KD cells were transduced with lentiviral vectors to express a doxycycline-inducible shRNA targeting HuR (shHuR). Western blot experiments revealed that HuR protein expression was significantly reduced 96 hours after doxycycline treatment at 2.5 μg/mL (Supplemental Figure 7A). HuR silencing in CFTR KD cells was associated with a significant decrease of Vav3 and fibronectin expression (Supplemental Figure 7A). To further validate the impact of HuR inhibition, we next treated polarized CTL and CFTR KD cells at ALI with CMLD-2 at 20μM. We measured the TEER 72 hours after treatment. TEER measured in polarized CFTR KD cells was significantly decreased in comparison with CTL cells (Figure 5A), confirming the epithelial integrity defect in CF. Interestingly, inhibition of HuR by CMLD-2 in the CFTR KD cells restored TEER to the same levels observed in CTL cells (Figure 5A). Decreased integrity of the CF airway epithelium is associated with Vav3-dependent aberrant formation of apical complexes rich in fibronectin and its β1 integrin receptor (6). We next tested if HuR inhibition could prevent Vav3 overexpression at the protein level. As shown by Western blot in Figure 5, B and C, CMLD-2 treatment of CFTR KD cells reduced Vav3 protein expression to control levels, as observed in CTL cells. In addition, preventing Vav3 overexpression with CMLD-2 decreased the expression of fibronectin and β1 integrin in CFTR KD cells (Figure 5, B and C). The effect of CMLD-2 on fibronectin expression was mediated by Vav3 inhibition, since fibronectin mRNA decay was not altered in CFTR KD cells (Supplemental Figure 6E). Finally, we show by confocal microscopy that targeting Vav3 mRNA stability by HuR inhibition markedly reduced apical fibronectin deposition in CFTR KD cells (Figure 5D). To confirm the absence of apical fibronectin deposition in presence of CMLD-2, we studied β1 integrin activity using 9EG7 antibody, which specifically recognizes the high-activity β1 integrin conformation (40). Interestingly, luminal β1 integrin activity was abolished (Figure 5D).

Among several small molecules, MS-444 has been identified in vitro and in vivo as an inhibitor of HuR cytoplasmic translocation (41–43). Since HuR cytoplasmic localization was increased in CFTR KD cells, we tested whether MS-444 would decrease Vav3 expression and, consequently, fibronectin apical deposition. Analysis of Vav3, Vav3.1 (Supplemental Figure 8, A and B, respectively), and fibronectin mRNA (Supplemental Figure 8C) by qPCR demonstrated that MS-444 treatment at 20 μM for 48 hours assisted in the prevention of their overexpression in CFTR KD cells. The inhibition of Vav3 (Supplemental Figure 9A) and fibronectin (Supplemental Figure 9A) overexpression in CFTR KD cells was confirmed at the protein level by Western blot. Importantly, the inhibition of HuR nucleocytoplasmic shuttling by MS-444 treatment prevented fibronectin deposition in polarized CFTR KD cells, as shown by confocal microscopy (Supplemental Figure 9B). These results suggest that restoring the posttranscriptional regulation of Vav3 mRNA by HuR inhibition improves the epithelial integrity and prevents fibronectin deposition at the luminal side of the CFTR KD epithelium.

### HuR inhibition reduced Vav3 expression and fibronectin deposition in primary CF HAECs.

**Figure 6:**
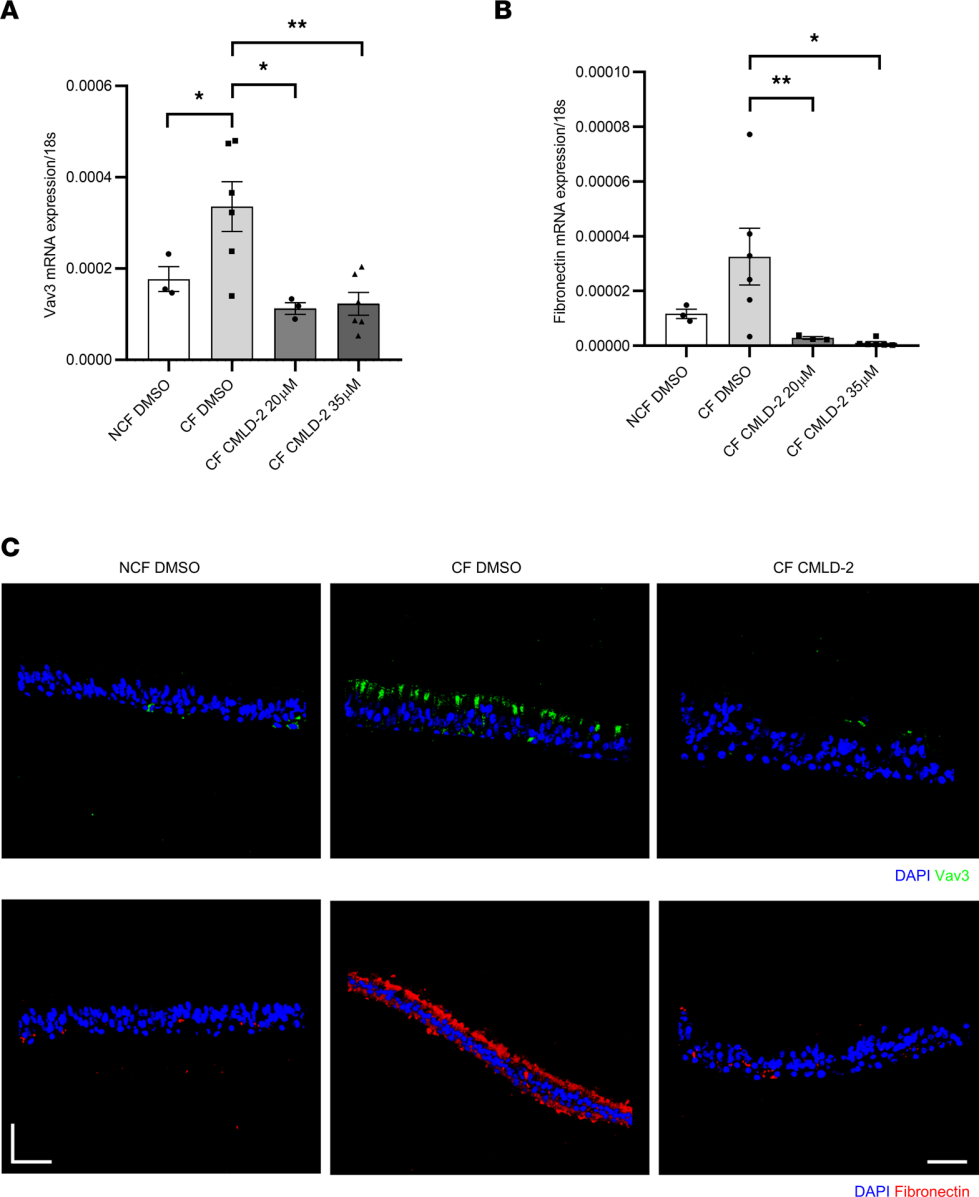
HuR inhibition restored Vav3 and fibronectin normal expression in CF primary HAECs. (**A** and **B**) Quantification of Vav3 (**A**) and fibronectin (**B**) mRNA expression by qPCR in CF versus NCF fully differentiated primary HAECs following CMLD-2 treatment. 18s served as an internal control. *N* = 3 donors in each group. Mann-Whitney *U* test, *P < 0.05, ** *P* < 0.01. (**C**) Representative confocal images of Vav3 (green) and fibronectin (red) immunostaining on cryosections of CF and NCF fully differentiated primary HAECs treated with CMLD-2. Nuclei are stained in blue. Scale bars: 40 μM.

To confirm this approach on a fully differentiated airway epithelium, we exposed primary CF HAECs grown at ALI to CMLD-2 at 20 μM or 35 μM. Vav3 and fibronectin expression and localization were analyzed 72 hours after treatment by qPCR and immunostaining, respectively. First, we confirmed that Vav3 and fibronectin were overexpressed at the mRNA (Figure 6, A and B, respectively) and protein level (Figure 6C) in CF HAECs, as compared with the NCF cells. Similar effects were observed for both CMLD-2 concentrations. Second, we observed a clear ectopic expression of Vav3 at the apical part of the CF epithelium, which was associated with an increased luminal fibronectin deposition (Figure 6C). Interestingly, CMLD-2 prevented Vav3 overexpression and inhibited deposition of fibronectin at the surface of the CF epithelium. We also tested the impact of MS-444 treatment at 20 μM on Vav3 and fibronectin expression in primary CF HAECs. Again, Vav3 and fibronectin mRNA expression was significantly decreased 72 hours after treatment (Supplemental Figure 10, A and B, respectively). These transcriptional effects were confirmed at the protein level by confocal microscopy (Supplemental Figure 10C). Furthermore, Vav3 inhibition by MS-444 decreased fibronectin luminal accumulation in primary CF HAECs.

HuR contains AREs in the 3′UTR of its mRNA, and there is evidence that HuR may regulate the stability of its own transcript (44). In keeping with this report, we found that CMLD-2 decreased the expression of HuR mRNA in primary CF HAECs (Supplemental Figure 11A), supporting that the effect of CMLD-2 on primary CF HAECs is mediated by its capacity to inhibit HuR/AREs interaction. This observation was also confirmed in CFTR KD cells (Supplemental Figure 11B). These results strengthen the role of HuR overexpression in Vav3/fibronectin accumulation in CF airway epithelial cells.

### HuR inhibition prevented Pa adhesion to CFTR KD cells.

**Figure 7:**
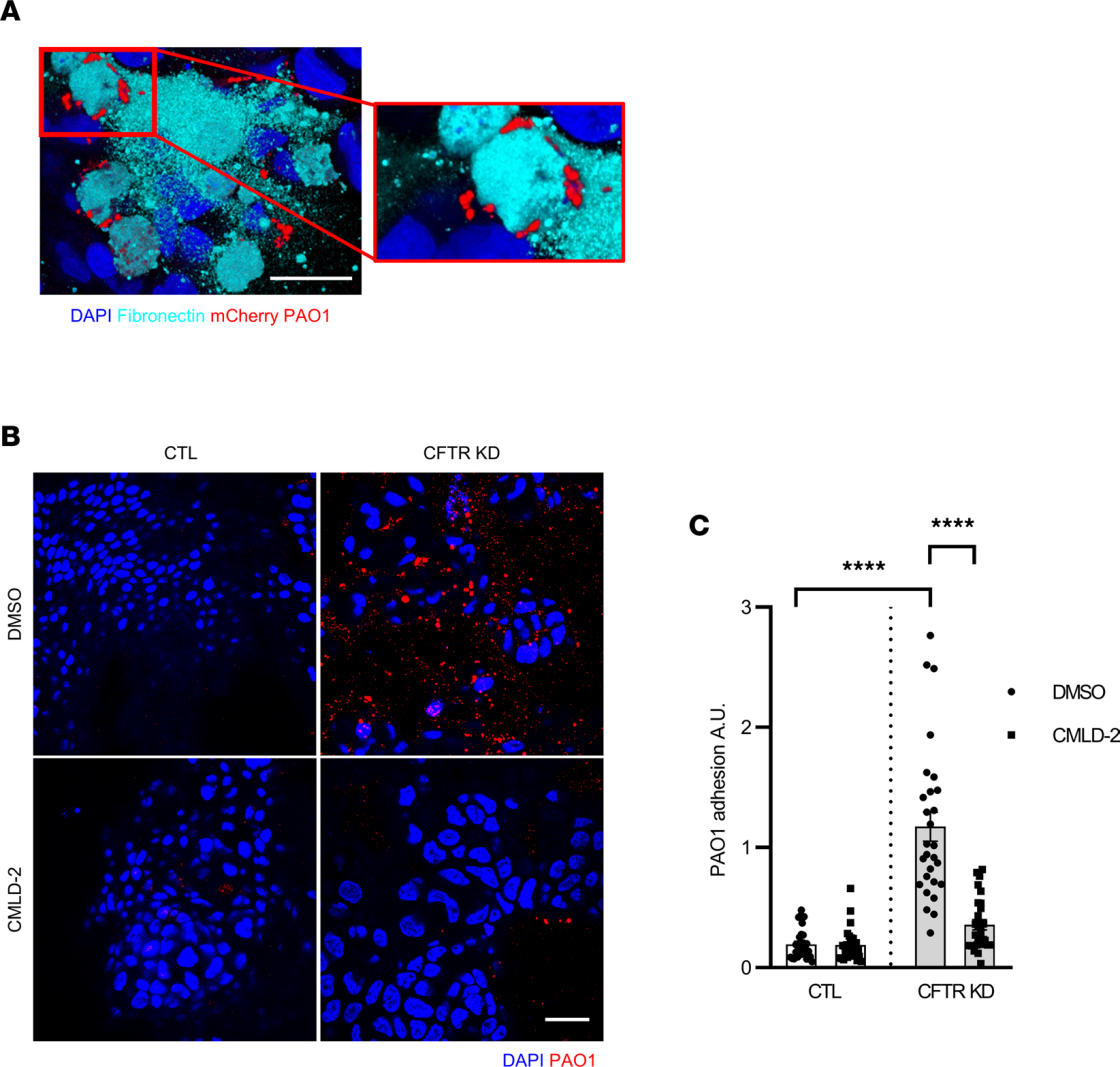
Targeting HuR inhibited *Pa* adhesion to the apical surface of CFTR KD epithelium. (**A**) Confocal microscopy analysis of fibronectin (cyan) and PAO1-expressing mCherry (red) localization in polarized CFTR KD Calu-3 cells at ALI. Right panel shows top view of representative images from 3D reconstruction of *Z* stack data. (**B** and **C**) *Pa* adhesion to the CFTR KD versus CTL Calu-3 cells following CMLD-2 treatment. Polarized Calu-3 cells were apically infected with 1 ***×*** 10^5^ CFU of PAO1 at 37°C in 5% CO_2_ atmosphere. One hour after infection, the adherent *Pa* strains were detected by immunostaining (**B**) and quantified using ImageJ (**C**). Top view of representative images from 3D reconstruction of *Z* stack data are shown, along with quantification. *n* = 3 in each group. Two-way ANOVA, ****P < 0.0001. Scale bars: 20 μM.

*Pa* adherence to fibronectin is critical for bacterial colonization and infection (7). Indeed, CFTR KD cells infected with PAO1-expressing mCherry confirmed the close interaction between fibronectin and *Pa* at the luminal side of the CF epithelium 1 hour after infection (Figure 7A). HuR/AREs interaction could represent a promising antiadhesive strategy against *Pa* infection in CF. To determine the efficiency of HuR inhibition on *Pa* adhesion to the CF airway epithelial cells, CTL and CFTR KD cells pretreated or not with CMLD-2 at 20 μM were apically infected with 1 ***×*** 10^5^ CFU of *Pa* strain PAO1. At 1 hour after infection, the adherent PAO1 was analyzed by immunostaining using a specific antibody against *Pa* (Figure 7B). Quantification confirmed increased PAO1 adhesion to CFTR KD cells as compared with CTL cells (Figure 7, B and C). Importantly, HuR inhibition by CMLD-2 prevented PAO1 adhesion to CFTR KD cells as compared with the nontreated condition (Figure 7, B and C). These results indicate that HuR overexpression causes *Pa* adhesion to the CF airway epithelium by stabilizing Vav3 mRNA, which leads to the formation of Vav3-dependent adhesion platforms rich in fibronectin at the apical surface.

## Discussion

We recently reported that the CF airway epithelium exhibits luminal bacterial docking platforms rich in fibronectin, which promote *Pa* adhesion. This phenotype was induced by an increased expression of the GEF Vav3 underneath the apical membrane of CF airway epithelial cells (6). In this study, we report that increased Vav3-dependent *Pa* adhesion to the CF airway epithelium is due to defective posttranscriptional regulation of Vav3. We report that the RBP HuR is overexpressed in F508del-CFTR and CFTR KD airway epithelial cells. We further show that HuR interacts with Vav3 mRNA, increases its stability, and thereby promotes Vav3 overexpression in CF. Furthermore, we demonstrate that targeting HuR-Vav3 mRNA interaction prevents *Pa* adhesion to the CF epithelium via inhibition of luminal fibronectin deposition.

RNA binding proteins play a major role in posttranscriptional control of RNAs (45). HuR is a RBP that generally stabilizes target transcripts and promotes mRNA translation (46, 47). Under normal conditions, HuR is generally localized in the nucleus, and its translocation to the cytoplasm can be observed in response to multiple stimuli. For instance, stress exposure promotes HuR nucleocytoplasmic shuttling to stabilize target mRNAs (48). Interestingly, we observed accumulation of HuR in the cytoplasm of F508del-CFTR and CFTR KD epithelial cells even without any extracellular stress exposure. Other studies reported HuR overexpression and translocation from the nucleus to the cytoplasm in lung tissues of smoker individuals, patients with COPD (49), patients with idiopathic pulmonary fibrosis, and mouse models of pulmonary fibrosis (50). The mechanism underlying HuR overexpression is still unknown. Since HuR mRNA contains AREs, a feedback loop has been proposed for HuR protein autoregulation of HuR mRNA. While HuR in the cytoplasm stabilizes its mRNA, nuclear accumulation of the RBP will favor HuR mRNA degradation. This mechanism was described in the mouse and *Drosophila* through alternative 3′ end formation of HuR mRNA (44, 51). Although our findings point to a potential autoregulation process, future research is needed to explore the mechanism regulating human HuR 3’UTR; such research may help researchers understand how CFTR mutation or knockdown can lead to HuR overexpression. HuR activity can be compensated by TTP, which is a major binding competitor of HuR that promotes mRNA degradation. Interestingly, we found that TTP is decreased in primary CF HAECs, confirming a posttranscriptional imbalance toward higher mRNA stability. Reduced TTP expression was previously observed in primary CF airway epithelial cells and the IB3-1 CF cell line (52). Indeed, these observations were recapitulated after CFTR knockdown in Calu-3 airway epithelial cells grown at ALI. Additionally, HuR overexpression was also observed in HeLa cells stably expressing G551D-CFTR, indicating that the posttranscriptional gene regulation imbalance was associated not only with defect in CFTR expression and trafficking, but also with gating of the anion channel. Thus, aberrant HuR/TTP expression is a consequence of CFTR dysfunction. An aberrant HuR expression and localization has been observed in lung and colorectal cancers, promoting tumorigenesis and poor prognosis of these patients (43, 53–55). The improvement of CF management and the increase of the life expectancy of CF patients have revealed the appearance of concomitant malignancies (56). Indeed, CFTR expression was negatively correlated with the prognosis of patients with lung cancer (57, 58), and metaanalysis confirmed that adults with CF had 5–10 times higher risk to develop colorectal cancer than the general population (59). In this context, it would be interesting to investigate if CFTR dysfunction–induced HuR overexpression may contribute to the predisposition of CF patients to carcinogenesis.

Only 8% of mRNAs express AREs regions that favors the recruitment of the deadenylases complex leading to mRNA decay (60). HuR binds to AREs at the 3′UTR region to competitively prevent the fixation of the deadenylases complex and consequently stabilize the bound mRNA target (48, 61). Multiple functional classes of AREs are present at the Vav3 mRNA 3′UTR, and it has been reported that HuR shows high affinity for motifs rich in uridine (62). Xie and collaborators describe a potential role of HuR in Vav3 mRNA stability by measuring Vav3 expression after HuR silencing in gastric cancer cell lines (18). Here, we demonstrate the physical interaction between Vav3 mRNA and HuR by combining the analysis of Vav3 3′UTR sequence, CLIP-Seq databases, RNP-IP, and RNA-Seq following HuR pull-down. Importantly, this interaction was enriched in CFTR KD cells, leading to higher Vav3 mRNA stability. The RNA-Seq data confirm that HuR binds to Vav3 mRNA at the 3′UTR. The reads mapping indicates that some RNA regions are recognized in both CTL and CFTR KD Calu-3 cells with higher signal when CFTR is knocked down. In addition, other sites were detected only in the CFTR KD cells, suggesting a stronger interaction. Future work using oligonucleotides to prevent the HuR-Vav3 mRNA interaction at the detected regions would be needed to confirm these observations. Vav3 transcripts can be alternatively spliced and consequently encodes for the transcript variant Vav3.1 (34). We confirmed that HuR interacts with both Vav3 isoforms. In addition to its involvement in mRNA stability and translation, HuR has been described as a regulator of the pre-mRNA alternative splicing. Thus, HuR may also regulate Vav3 alternative splicing. It has been reported that 80% of the binding sites recognized by TTP and HuR are overlapping. However, despite these similar binding preferences, a subtle mechanism whereby TTP preferentially binds to sequences with higher adenosine amount has been reported, while HuR has more affinity for motifs rich in uridine (36, 62). In agreement with these reports, analysis of the 3′UTR of the Vav3 transcript points to an enrichment in uridine. In addition, lentiviral expression of TTP was not able to alter Vav3 overexpression in CFTR KD cells. Although more data are needed to validate whether TTP overexpression or other TTP family members are functional (63), our results suggest that deregulation of Vav3 expression in CF airway epithelial cells is predominantly controlled by HuR.

Different strategies were used to investigate the causal relationship between HuR and Vav3 overexpression in CF cells. HuR is ubiquitously expressed with important influence on several cellular responses (64). Thus, to avoid side effects that could be induced by a constitutive knockdown, we used a Tet-On knockdown system based on a doxycycline-inducible shRNA. Whereas HuR expression was decreased by only 35% after 96 hours of doxycycline induction, this was sufficient to decrease Vav3 and fibronectin expression, confirming the role of HuR in Vav3 mRNA regulation in CFTR KD cells. Because of the low stability and delivery issues associated to siRNA therapy, pharmacologic inhibition of RBPs is considered as a promising targeting approach in comparison with HuR silencing (65). Therefore, we used pharmacological inhibitors with different mechanisms of action. MS-444 was identified by high-throughput screening as an inhibitor of HuR dimerization and, thus, its nucleocytoplasmic translocation (41). HuR overexpression in CF was associated by its cytoplasmic redistribution. Since HuR exerts its mRNA stability function through its cytoplasmic translocation, prevention of HuR shuttling to the cytoplasm with MS-444 also prevented fibronectin deposition by inhibiting Vav3 overexpression in CFTR KD Calu-3 cells and in primary CF HAECs. MS-444 was also described as an inhibitor of the myosin light chain kinase (66). To rule out any potential unspecific effect of MS-444 on fibronectin expression, we used CMLD-2, a cumarin-derived small molecule identified as an inhibitor of HuR interaction with its target mRNAs (35). Previous studies reported dose-dependent cytotoxicity of CMLD-2 in several cell lines, including the non–small cell lung cancer model (37). In this study, measurement of LDH, a well-established indicator of cell toxicity, demonstrated that CTL and CFTR KD Calu-3 cells were not affected by CMLD-2. We showed by RNP-IP and RNA-Seq that CMLD-2 treatment prevents the enriched HuR interaction with Vav3 and Vav3.1 mRNAs in CFTR KD Calu-3 cells. CMLD-2 also inhibited Vav3 overexpression in CFTR KD Calu-3 cells, likely because of reduced stability of Vav3 mRNA. Importantly, CMLD-2 inhibition of Vav3 overexpression prevented fibronectin expression in CFTR KD Calu-3 cells. The effect of CMLD-2 on fibronectin expression is mediated by Vav3 posttranscriptional regulation, since fibronectin mRNA stability was not affected in CFTR KD Calu-3 cells. Although additional transcriptomic analysis of CF airway epithelial cells treated with CMLD-2 is needed to identify other genes that are potentially stabilized by HuR, we believe that HuR overexpression might be associated with defective fibronectin remodeling. Fibronectin remodeling is a dynamic process with cycles of production and degradation regulated by the protease/antiprotease balance. For example, fibronectin cleavage is controlled by the matrix metalloprotease MMP-9, which is increased in CF patients (67). Importantly, there is ample evidence that HuR stabilizes MMP-9 transcript (68, 69), suggesting that HuR overexpression in CF might correlate with higher MMP-9 expression and activity, leading to aberrant fibronectin cleavage. It has also been described in several inflammatory diseases that HuR may regulate the expression of proinflammatory genes by stabilizing mRNAs of proinflammatory cytokines like TNF-α, IL-6, and IL-8 (70–72). However, we did not observe changes on basal IL-6 and IL-8 mRNA expression in CFTR KD Calu-3 cells treated with CMLD-2. Whether HuR may contribute to the inflammatory response of CF cells remains to be further investigated. For example, the expression of additional genes known to be regulated by HuR, such as cyclooxygenase-2 (73), could be monitored during infection or after stimulation with bacterial virulence factors.

Infection is a multifactorial process that involves close interaction between the bacteria, the host, and the environment. In CF, the irreversible attachment of *Pa* to the epithelium is promoted by deposition of fibronectin on the airway epithelium surface, as visualized by the colocalization between mCherry-tagged *Pa* and fibronectin at the apical side of CFTR KD cultures. This interaction is mediated by at least 6 porins expressed at the outer membrane of *Pa* that were identified as fibronectin-binding proteins (74). We show here that reducing ectopic fibronectin expression by CMLD-2 treatment efficiently prevented *Pa* adhesion to the CFTR KD airway epithelium surface. Importantly, inhibition of HuR-Vav3 mRNA was also able to inhibit fibronectin deposition in fully differentiated primary CF HAECs. It has been reported that targeting cellular fibronectin reduced *Pa* adhesion to explant outgrowth cultures of dedifferentiated nasal epithelial cells (75). Therefore, CMLD-2 treatment could represent a promising antiadhesive approach in CF. In addition to the antiadhesive effect, CMLD-2 increased the TEER of polarized CFTR KD Calu-3 cells, indicating that HuR inhibition strengthens the airway epithelial integrity. Accordingly, a clear relationship between the epithelial integrity and posttranscriptional regulation have been described in the intestine through the regulation of the small Rho GTPase Cdc42 by HuR. Importantly, Vav3 represents a Rho GTPase activator that is critical in the regulation of the actin cytoskeleton network. Moreover, Vav3 silencing improved the CF epithelial integrity by controlling the cytoskeleton organization through Cdc42 (6). Thus, targeting HuR-Vav3 mRNA interaction improves the barrier function of the CF airway epithelium and prevents *Pa* adhesion.

Although further investigations are needed on the impact of HuR inhibition on *Pa* growth, biofilm formation, and antibiotic resistance in HAEC models, our results demonstrate that targeting the HuR pathway represents an attractive therapeutic approach, especially in a context of increasing *Pa* resistance to antibiotics and, thus, is vital to propose alternative and complementary treatment strategies in order to improve the clinical outcomes of CF patients. It is remarkable that HuR overexpression is observed for different classes of CFTR mutations, therefore representing a common target, whereas pharmacological therapies with CFTR modulators are limited to specific CFTR mutations.

## Methods

### Experimental model and subject details.

Primary HAECs were purchased from Epithelix Sàrl (Switzerland). These cells were isolated from bronchial biopsies and were cultured on Transwell inserts in MucilAir Culture Medium from Epithelix Sàrl and differentiated at the ALI. The clinicopathological characteristics of the NCF and CF donors are provided in the Supplemental Table 1. The airway epithelial cell line Calu-3 was purchased from the American Type Culture Collection (ATCC HTB-55) and cultured in Minimum Essential Medium (MEM) GlutaMAX (Thermo Fisher Scientific) supplemented with 1% nonessential amino acids (NEAA, Bioconcept) 100×, 1% HEPES 1M, 1% sodium pyruvate 100×, 10% heat-inactivated FBS, and penicillin/streptomycin/amphotericin B (Bioconcept) at 37°C in humidified 5% CO_2_ atmosphere. Calu-3 cells were seeded on Petri dishes or coverslips for monolayers, while polarized Calu-3 cells were obtained by seeding 175,000 cells in 0.33 cm^2^ surface/0.4 μm pore polyester membrane Transwell inserts (Costar) in liquid-liquid interface for 5 days. At 100% of confluency, the Calu-3 cells were polarized at ALI for at least 15 days. HeLa cells expressing WT-CFTR or G551D-CFTR were cultures in DMEM high glucose L-glutamine (Thermo Fisher Scientific) containing 1% NEAA 100×, 1% HEPES 1M, 10% heat-inactivated FBS, and penicillin/streptomycin/amphotericin B at 37°C in humidified 5% CO_2_ atmosphere.

TTP overexpression in Calu-3 cells was obtained using lentiviral particles. Briefly, the cells were seeded at 6 × 10^4^ and infected with the control lentiviral vector, pCLX-Ubi-GFP, or the vector coding for TTP, pCWPGKBSD-TTP, at a multiplicity of infection (MOI) of 1 in the presence of Polybrene (Sigma-Aldrich) for 4 hours at 37°C in a humidified 5% CO_2_ atmosphere. This step was followed by Blasticidin selection. pCLX-UBI-GFP (Addgene plasmid 27245; http://n2t.net/addgene:27245; RRID: Addgene_27245) and pCWPGKBSD-TTP were gifts from Patrick Salmon. HuR silencing in CFTR KD Calu-3 cells was obtained using the Tet-On inducible expression system. In total, 6 × 10^4^ CFTR KD Calu-3 cells were seeded on Petri dishes and infected with the lentiviral vector carrying the doxycycline-inducible shHuR plasmid (Tet-pLKO.puro_shHuR CDS) in the presence of hexadimethrinbromid (Sigma-Aldrich) for 4 hours at 37°C in humidified 5% CO_2_ atmosphere. Following puromycin selection, the transduced cells were treated with doxycycline for 48 hours or 96 hours at 2.5 μg/mL. Tet-pLKO.puro_shHuR CDS was a gift from Sandra Martha Gomes Dias (Addgene plasmid 110411; http://n2t.net/addgene:110411; RRID: Addgene_110411).

### qPCR.

RNAs were isolated with RNeasy mini kit (Qiagen) following the manufacturer’s instructions. The quality and the quantity of the extracted RNA was verified by the spectrophotometer Nanodrop 2000 (Thermo Fisher Scientific). Before the reverse transcriptase PCR (RT-PCR), the extracts were purified from Genomic DNA with gDNA wipeout buffer for 2 minutes at 42°C, and complementary DNA was synthetized with the QuantiTect Reverse Transcription Kit (Qiagen). qPCR experiments were performed with StepOnePlus Real-Time PCR system using the PowerUp SYBR Green Master Mix. mRNA expression is represented as absolute value (2^–ΔCt^) normalized to 18s expression. All the primers were purchased from Microsynth AG, and the specific sequences for each gene are given in the Supplemental Table 2.

### Western blot.

Proteins were extracted using Nonidet-P40 lysis buffer (150 mM sodium chloride, 50 mM Tris [pH 8.0], 1% NP-40 (Applichem), and Roche cOmplete Protease Inhibitor Cocktail). After 15 minutes of centrifugation at 14,000*g* at 4°C, the protein concentration was determined with Pierce BCA Protein Assay Kit (Thermo Fisher Scientific). In total, 10 μg or 20 μg of proteins were separated by electrophoresis in SDS polyacrylamide gel. The proteins were then transferred into the nitrocellulose membrane Macherey-Nagel NCP Porablot Membrane and incubated for 1 hour at room temperature in 3% BSA in PBS-Tween (0.01%) buffer. Next, the membrane was incubated overnight at 4°C with the antibodies listed in the Supplemental Table 3 with agitation. All the loadings were controlled with Ponceau S staining and anti-GAPDH antibody. When the primary antibodies are fixed, the membrane were rinsed with PBS-Tween (0.01%) buffer and followed by horseradish peroxidase–coupled (HRP-coupled) secondary antibody incubation. Proteins were finally revealed by detecting of HRP with the SuperSignal West Pico PLUS chemiluminescent substrate (Thermo Fisher Scientific). The chemiluminescent signal was captured by the Syngene PXi image analysis system and analyzed using Quantity One Analyses Software (Bio-Rad).

### Subcellular fractionation.

To separate the nuclear and the cytoplasmic fractions, proteins were extracted from Calu-3 cells grown as monolayers or polarized at ALI with 500 μL of a subcellular fractionation buffer (20 mM HEPES, 10 mM KCl, 2 mM MgCl_2_, 1 mM EDTA, 1 mM EGTA, 1 mM DTT and Roche cOmplete Protease Inhibitor Cocktail). After 15 minutes on ice, the cell suspension was collected by scraping and was passed through a 27-gauge needle 10 times. After 20 minutes on ice, the cell lysate was centrifuged at 850*g* at 4°C for 5 minutes. This step separates the cytoplasmic fraction present in the supernatant from the pellets that contain the nuclei. Next, the nuclear pellets were washed with 500 μL of the subcellular fractionation buffer, centrifuged at 850*g* at 4°C for 10 minutes, resuspended in TBS with 0.1% SDS, and sonicated on ice. The purity of both cellular fractions was determined using the fraction WB antibody cocktail (Abcam).

### Immunofluorescence.

Cryosections from Tissue-Tek O.C.T. embedded CF and NCF primary HAECs were immediately fixed in 4% paraformaldehyde solution (PFA) for 30 minutes on ice and permeabilized for 15 minutes at room temperature with 0.1% Triton 100× buffer. Polarized CFTR KD and CTL Calu-3 cells treated or not with CMLD-2 (Sigma-Aldrich) or MS-444 (MedChemExpress) were fixed with 4% PFA for 20 minutes at room temperature and permeabilized for 15 minutes at room temperature with 0.1% Triton 100× buffer. The nonspecific sites were blocked by PBS-BSA 1% solution for 30 minutes at room temperature, and the slides were then incubated with primary antibody recognizing the proteins of interest at 4°C overnight. After several PBS washing, the target proteins were detected by mean of secondary anti-rabbit, anti-mouse, or anti-rat secondary Alexa Fluor 488, 568, or 647 antibodies for 1 hour at RT (Supplemental Table 3). DAPI counterstaining was used to visualize the nuclei. The images and *Z* stacks were obtained with LSM700 confocal microscope and ZEN software (ZEISS). The images were analyzed using ZEN, ImageJ (NIH), and IMARIS Microscopy Imaging Software.

### RNP-IP.

For RNP-IP experiments, Calu-3 cells treated or not with CMLD-2 were seeded on 60 mm culture dishes. At 70%–80% confluence, RNP complexes were isolated using polysome lysis buffer (20 mM Tris HCl [pH 7.5], 5 mM MgCl_2_, 150 mM NaCl, 1 mM DTT, 0.05% NP-40, Roche cOmplete Protease Inhibitor Cocktail, RiboLock RNase Inhibitor [100 units]). The cell lysates were centrifuged at 10,000*g* for 15 minutes at 4°C, and again, the protein concentration was quantified with Pierce BCA protein assay kit. Next, a preclearing step was performed by incubating 800 μg of the extracted proteins with 50 μL of the Protein A/G magnetic beads mix Pureproteome at 4°C. Two hours after incubation, the supernatant was collected and incubated with anti-HuR specific antibody (IP HuR), anti–flag IgG rabbit antibody (IgG), or without any antibody (NoIP) in presence of 1 μL of DTT (1M), 40 units of RiboLock RNase Inhibitor, and 33 μL of EDTA (0.5M) overnight at 4°C. To immunoprecipitate the complex HuR/mRNA, 50 μL of protein A/G magnetic beads mix Pureproteome were added to the mix (protein and antibody) for 1 hour at 4°C. Then, the beads are collected using a magnetic rack, washed with NT2 buffer (50 mM Tris-HCl, 150 mM NaCl, 1 mM MgCl_2_, 0.05% NP-40) at 4°C and finally incubated with RLT buffer. The RNA bound to HuR was isolated using RNeasy mini kit (Qiagen).

### RNA-Seq.

The transcriptomic profile of the RNA extracted after HuR pull-down was analyzed by RNA-Seq. RNP-IP was performed on CTL and CFTR KD Calu-3 cells treated or not with CMLD-2. The NoIP condition (without anti-HuR antibody) was used as a negative control. The quality control of the RNA samples following the RNP-IP was verified using the 2100 Bioanalyzer system (Agilent Technologies). Next, the ribosomal RNA was depleted with the Ribo-Zero Plus rRNA Depletion kit (Illumina). RNA-Seq was performed using the sequencer Illumina HiSeq 4000 by the *iGE3* Genomic Platform at the Faculty of Medicine, University of Geneva. The fastq files were mapped to the UCSC human genome with STAR v.2.7.0f. The biological QC was performed with picard tools. The table of counts with the number of reads mapping to each gene feature of UCSC hg38 human reference was prepared with HTSeq v0.9.1 (htseq-count). After normalization, the poorly detected genes were filtered out, and 15,450 genes with a count above 10 were kept for the analysis. The 3′UTR mapping of Vav3 mRNA was performed using the Integrative Genomics Viewer (IGV) software.

### 3′UTR region analysis.

The identification of the AREs motifs in the 3′UTR region of Vav3 mRNA was retrieved from the public database AREsite2 (31) (http://rna.tbi.univie.ac.at/AREsite). The RBP binding sites on the 3′UTR region of Vav3 mRNA were identified by CLIP-Seq technology analyzed by the peak calling method Piranha and were collected from the POSTAR3 database (33) (http://postar.ncrnalab.org).

### mRNA stability assay.

CFTR KD and CTL Calu-3 cells were seeded in a 6-well plate at a density of 250,000 cells. After 48 hours, the cells were treated or not with actinomycin-D (Sigma) at 5 μg/mL for 4, 6, and 8 hours. Next, the cells are washed with PBS, and the total RNA was extracted using the RNeasy mini kit (Qiagen).

### LDH cytotoxicity assay.

Cellular cytotoxicity was measured using CyQUANT LDH assay (Thermo Fisher Scientific). Briefly, CFTR KD and CTL Calu-3 cells were seeded in a 96-well plate in 2 sets. The first set was intended to measure the maximum LDH release by adding a 10× lysis solution for 45 minutes at 37°C, while the second set was used to measure the spontaneous LDH release. In total, 50 μL of each sample was transferred to a new 96-well plate, and 50 μL of the reaction mixture was added to each sample. The mix was incubated for 30 minutes at room temperature in the dark. Afterward, the reaction was stopped by adding 50 μL of the Stop Solution (Thermo Fisher Scientific) to each sample for 1 hour, and the absorbance was then measured at 490 nm and 680 nm. First, the optimum cell number for this assay was determined by measuring the maximum and spontaneous LDH release on a serial dilution of cells from 0 to 10,000 cells. Next, 5,000 CFTR KD and CTL Calu-3 cells were plated on a 96-well plate in 3 sets. After overnight incubation at 37°C in humidified 5% CO_2_ atmosphere, different concentrations of CMLD-2 were added to the culture media for 24 hours. Finally, the spontaneous, the maximum, and the CMLD-2–induced LDH activity were measured as described above.

### TEER.

To measure the TEER, CFTR KD and CTL Calu-3 cells were grown on Transwell inserts, polarized at ALI, and treated with CMLD-2 for 72 hours. After PBS washes, the TEER was measured by the epithelial voltmeter (EVOM, World Precision Instruments Inc.) according to ref. 29. Resistance measurements were performed in duplicate for each filter and expressed as Ω·cm^2^.

### Pa adhesion assay.

*Pa* adhesion was studied using the laboratory strains PAO1 or PAO1-expressing mCherry that were grown in Lysogeny broth (LB) medium. Twenty-four hours before PAO1 infection, the culture medium of the polarized CFTR KD and CTL Calu-3 cells was replaced by an antibiotics-free medium. Next, the Calu-3 cells treated with CMLD-2 for 72 hours were infected apically with a final inoculum of 1 × 10^5^ CFU of PAO1 or 2 × 10^6^ CFU of PAO1-expressing mCherry and incubated at 37°C in 5% CO_2_ atmosphere. One hour after infection, the cells were rinsed with PBS and fixed with PFA for 30 minutes at room temperature. After the permeabilization step, adherent PAO1 bacteria were detected using *Pa*-specific antibody, while PAO1-expressing mCherry bacteria were visualized in combination with fibronectin immunostaining. *Pa* adhesion was analyzed by confocal imaging and quantified using ImageJ software.

### Data availability statement.

The data set for this article, including Binary Alignment Map files from the RNA-Seq, can be found at https://doi.org/10.26037/yareta:iwumbxaipvehvjzss4sj3wmcy4 The data set will be preserved for 10 years.

### Statistics.

Values are represented as mean ± SEM. The symbol *N* represents the number of donors form which primary HAECs were isolated and the symbol *n* represents the number of replicates. The statistical tests were realized using GraphPad Prism 8.0 or SigmaStat softwares. The differences between 2 groups were analyzed by Student’s *t* test or the nonparametric Mann-Whitney *U* test. Two-way ANOVA was performed to examine the differences between more than 2 groups. *P* < 0.05 was considered significant.

## Author contributions

M. Badaoui and MC conceived and planned the study design. CS and M. Badaoui designed RNP-IP assays. AL and TK designed infection experiments. M. Badaoui, AL, and M. Bacchetta carried out experiments. M. Badaoui, CS, TK, CVD, MF, and MC participated to analyses and discussion. M. Badaoui and MC wrote the manuscript. MC contributed to supervision, project administration, and funding acquisition. All authors reviewed the manuscript.

## Supplementary Material

Supplemental data

## Version 1 01/05/2023

In-Press Preview

## Version 2 02/08/2023

Electronic publication
