# Supplementary material for: Targeting HuR-Vav3 mRNA interaction prevents Pseudomonas aeruginosa adhesion to the cystic fibrosis airway epithelium
Source: JCI Insight. 2023 Feb 8;8(3):e161961. doi: 10.1172/jci.insight.161961 (PMC9977432; doi:10.1172/jci.insight.161961)
Supplement: Supplemental data [file jciinsight-8-161961-s159.pdf]

Targeting HuR-Vav3 mRNA interaction prevents *Pseudomonas aeruginosa* adhesion to the cystic fibrosis airway epithelium

Mehdi Badaoui[1], Cyril Sobolewski[1 #], Alexandre Luscher[2], Marc Bacchetta[1], Thilo Köhler[2], Christian van Delden[2], Michelangelo Foti[1], Marc Chanson[1,*]

[1] *Department of Cell Physiology & Metabolism, Faculty of medicine, University of Geneva, Switzerland*

[2] *Department of Microbiology & Molecular Medicine, Faculty of medicine, University of Geneva, Switzerland*

[#]*Present address: University of Lille, Inserm U1286 - INFINITE - Institute for Translational Research in Inflammation, F-59000 Lille, France*

[*]*Geneva Center for Inflammation Research (GCIR), Faculty of medicine, University of Geneva, Switzerland*

Corresponding author:

Marc Chanson, PhD

Department of cell physiology and metabolism

Faculty of Medicine (CMU)

1, Rue Michel-Servet, 1211 Geneva (Switzerland)

Tel: +41 22 37 95 206; **Fax:** +41 22 37 95 260

Email: Marc.Chanson@unige.ch

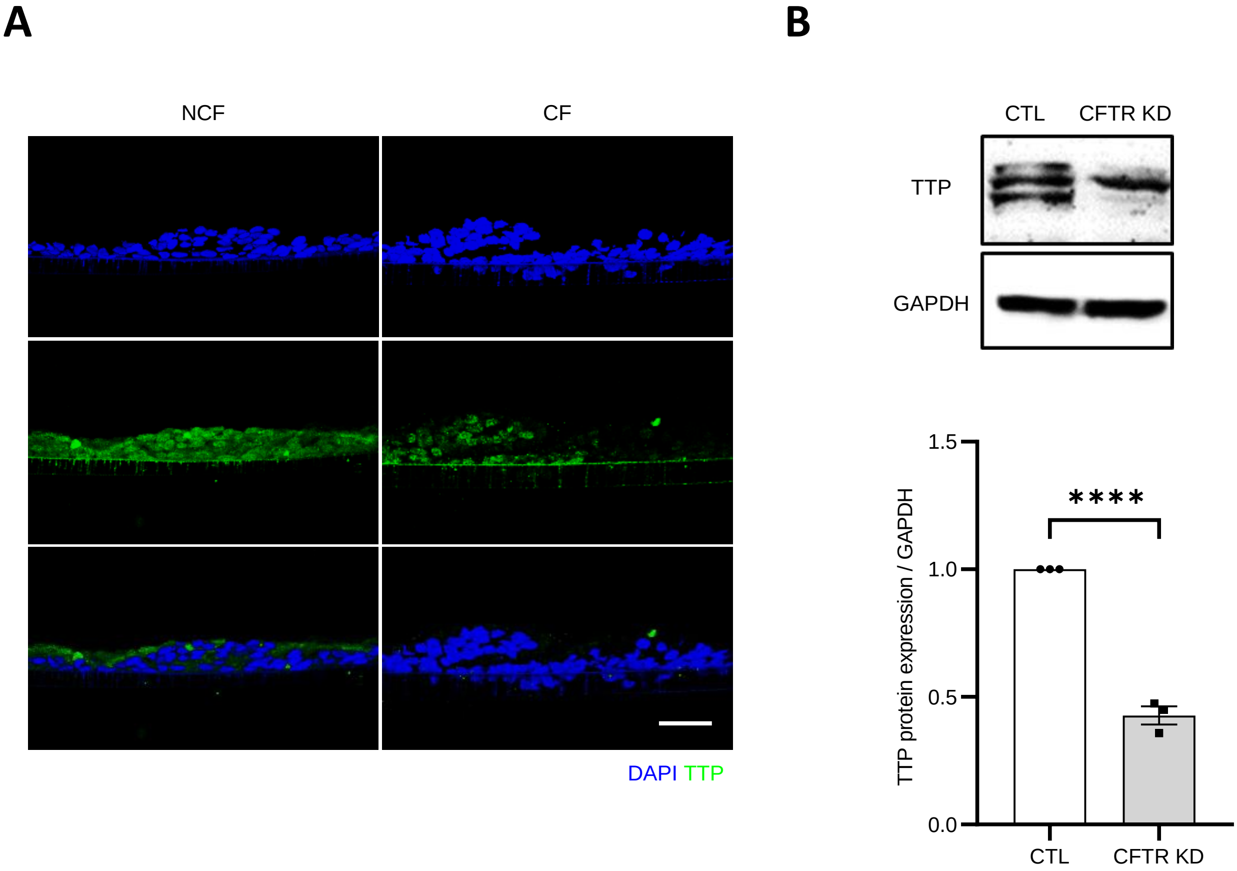

<u>Supplemental Figure 1.</u> TTP expression is decreased in primary CF HAECs and CFTR KD Calu-3 cells. **A-** Representative confocal images of TTP immunostaining (green) on cryosections of CF and NCF fully differentiated primary HAECs. Nuclei are stained in blue. Scale bar: 30µm. **B-** Representative Western blot showing TTP expression in CFTR KD cells *vs* CTL Calu-3 cells. GAPDH served as an internal control. The quantification is shown on the lower panel. n=3 in each group. *T*.Test, ****p<0.0001.

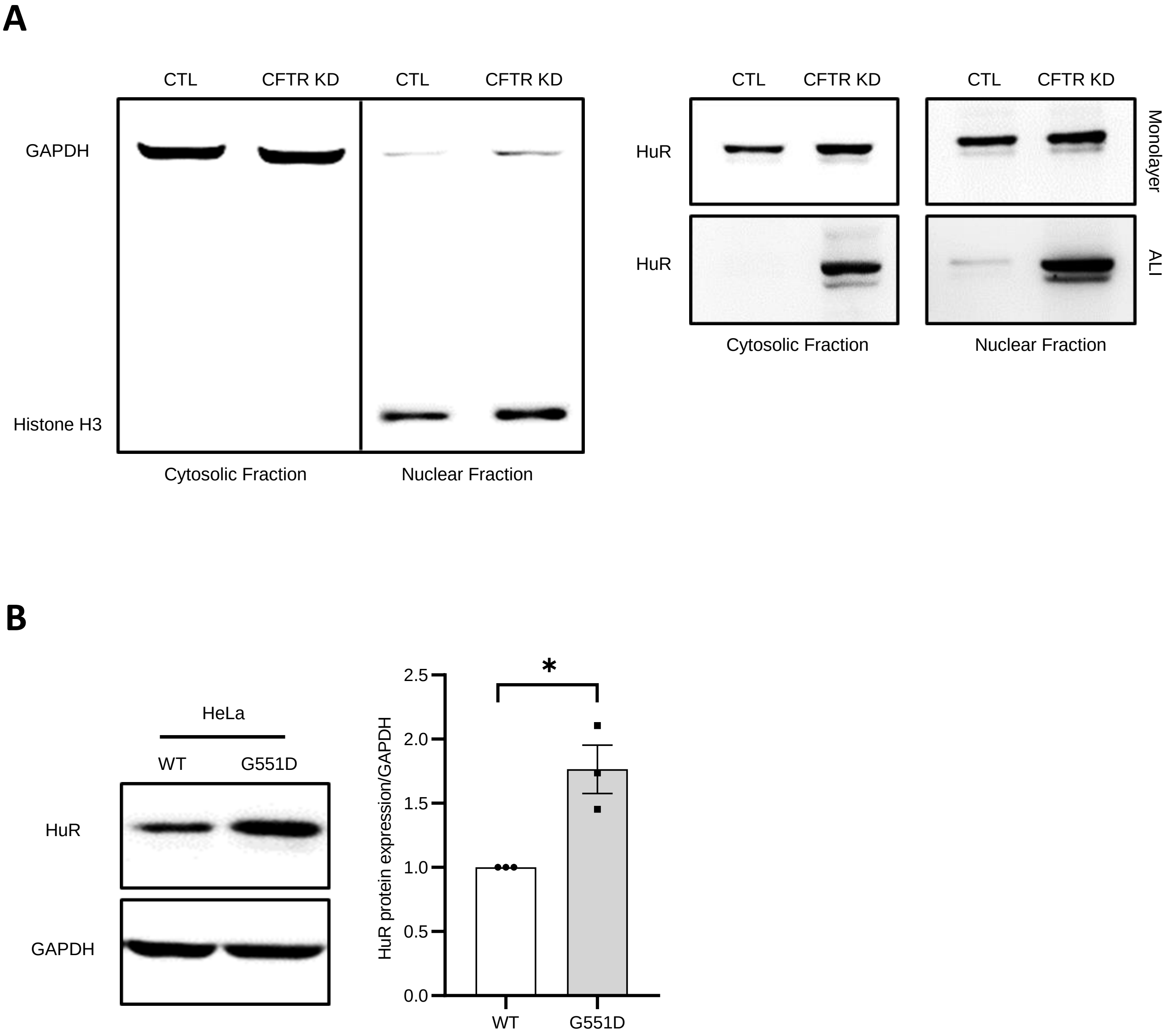

<u>Supplemental Figure 2.</u> **A**- Representative Western blot showing the purity of the cytosolic and the nuclear fractions using specific markers, respectively GAPDH and Histone H3. HuR expression in the different fractions was assessed by Western blot on Calu-3 cells grown as monolayer or polarized at ALI. **B**- Representative Western blot showing HuR expression in G551D-CFTR cells *vs* WT-CFTR HeLa cells. GAPDH served as an internal control. The quantification is shown on the right panel. n=3 in each group. *T*.Test, *p<0.05.

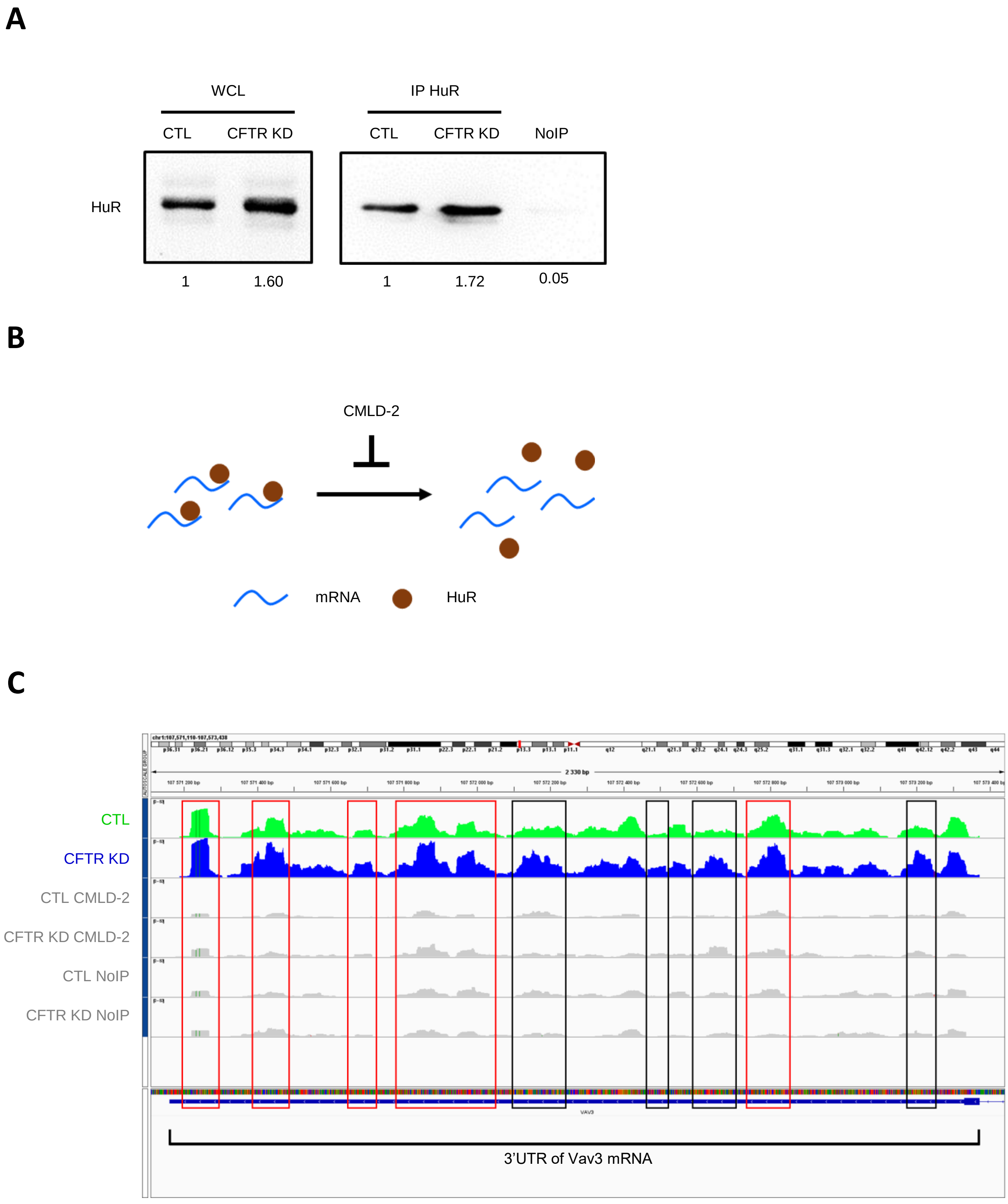

<u>**Supplemental Figure 3.**</u> **RNA immunoprecipitation sequencing after HuR pull-down. A-** Representative Western blot showing anti-HuR antibody specificity after HuR immunoprecipitation (IP HuR) in CFTR KD cells *vs* CTL Calu-3 cells. HuR expression in the Whole-Cell Lysates (WCL) is shown on the left panel. NoIP represents the negative control. **B-** Schematic showing the disruption of HuR interaction with the target mRNA by CMLD-2. **C-** Visualization of the RNAseq data by Integrative Genomics Viewer. The mapping of the 3'UTR region of Vav3 mRNA reveals the reads sequenced and their alignment across the 3'UTR as peaks. CTL cells after HuR pull-down are plotted in green while CFTR KD cells after HuR pull-down are plotted in blue. CMLD-2 treated cells and the NoIP conditions are shown in grey.

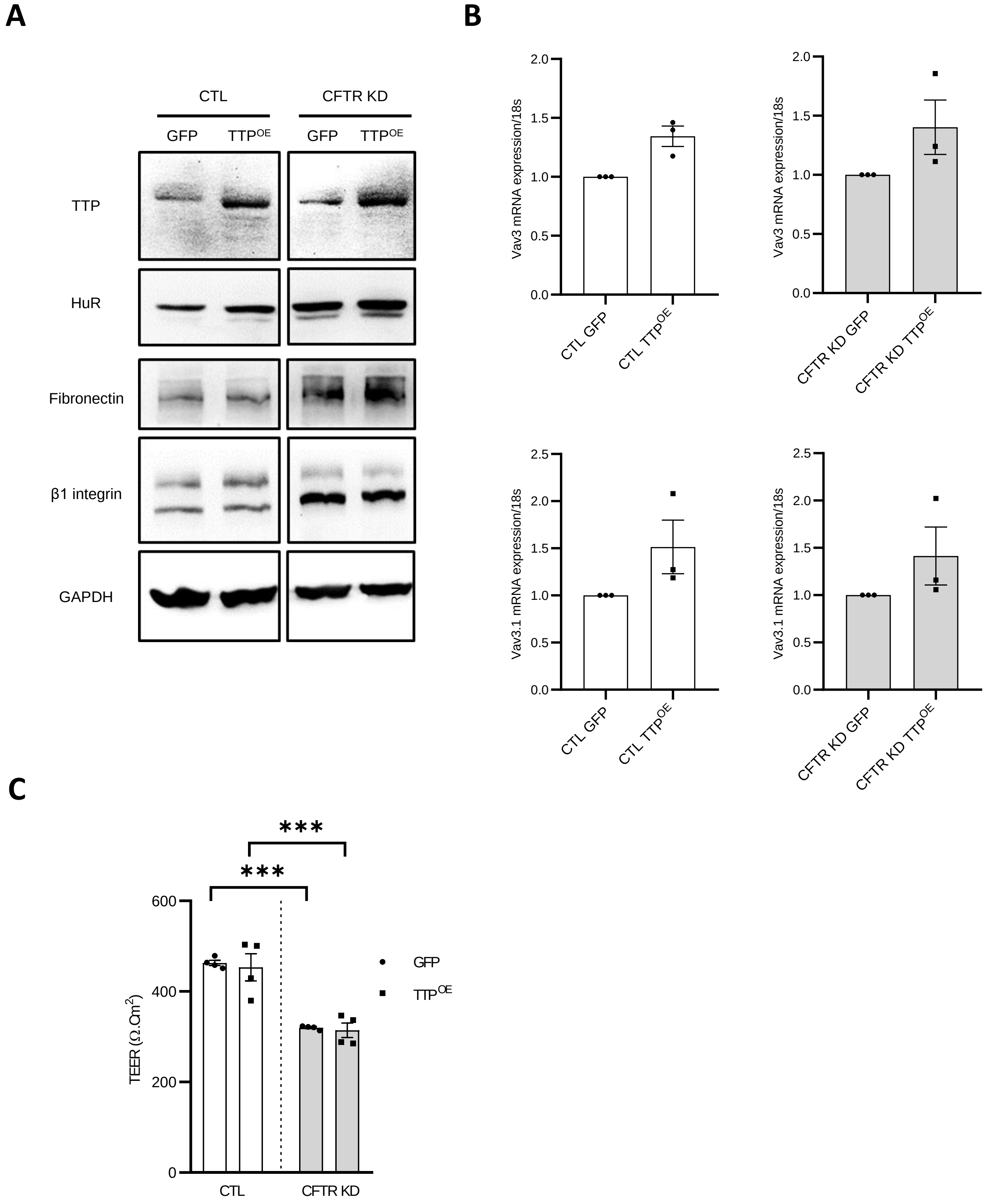

<u>Supplemental Figure 4.</u> Stable TTP overexpression in Calu-3 cells did not affect Vav3 regulation. **A-** Representative Western blot showing TTP, HuR, fibronectin and β1 integrin expression in CFTR KD cells *vs* CTL Calu-3 cells overexpressing or not TTP. GAPDH served as an internal control. **B-** Quantification of the relative mRNA expression of Vav3 and Vav3.1 by RT-qPCR in CFTR KD cells *vs* CTL Calu-3 cells overexpressing or not TTP. 18s served as an internal control. n=3 in each group. Mann-Whitney test, $p>0.05$. **C-** Transepithelial electrical resistance (TEER) measurements in CFTR KD cells *vs* CTL Calu-3 cells overexpressing or not TTP polarized at air-liquid interface. n=4 in each group. Two-Way ANOVA, ***$p<0.001$.

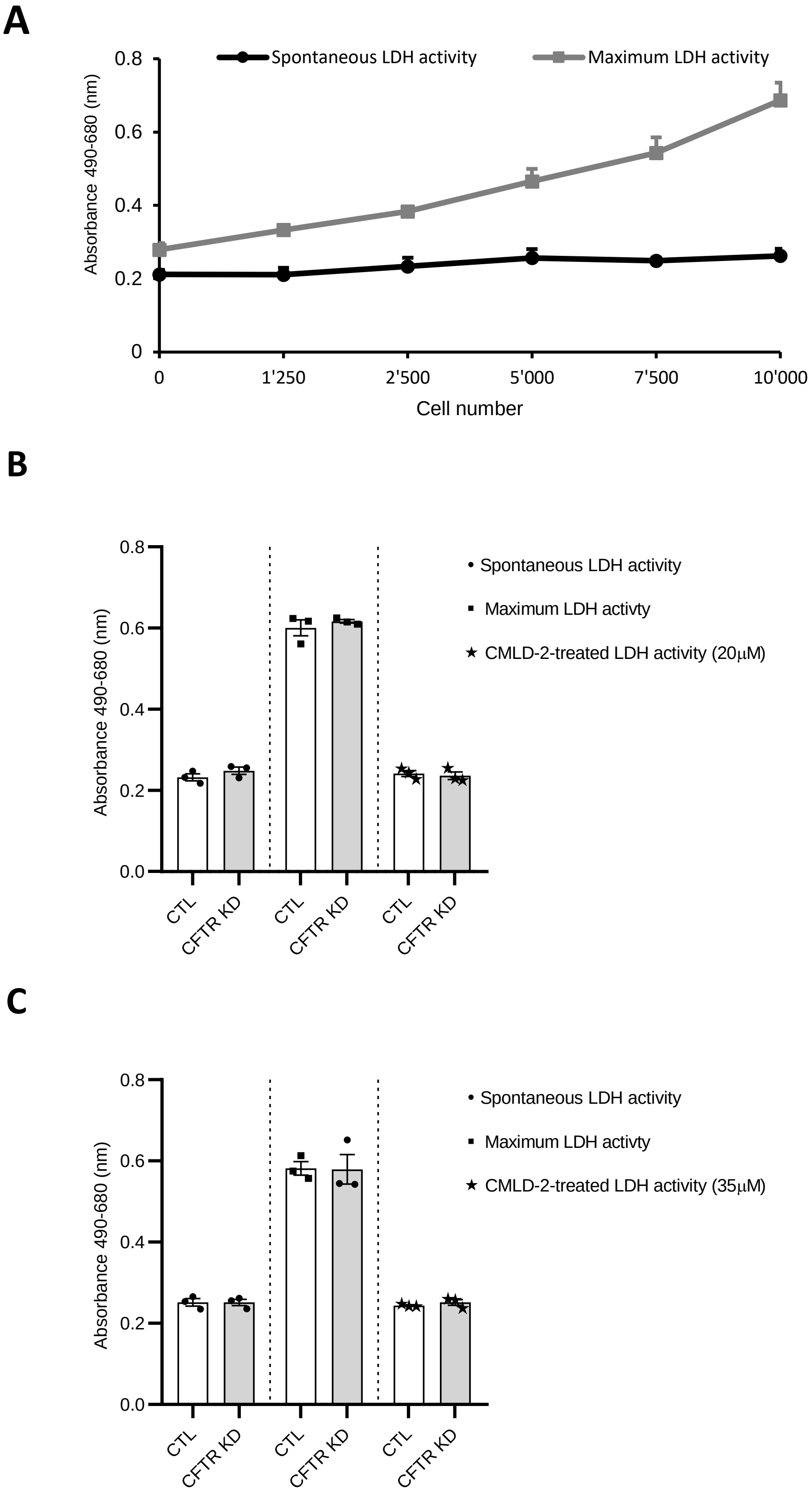

<u>Supplemental Figure 5.</u> Measurement of the cell cytotoxic in Calu-3 cells treated with CMLD-2. A- Calu-3 cells were seeded into a 96-well plate in two sets at different cell densities. The spontaneous LDH release was measured after water treatment, while the maximum LDH release was measured after treatment with 10X lysis solution. The LDH activity was determined by measuring the absorbance at 490nm and 680nm. **B, C-** CFTR KD cells and CTL Calu-3 cells were seeded into a 96-well plate in three sets. The spontaneous and maximum LDH release were measured as described above. CMLD-2-induced cytotoxicity was measured after treated with 20µM (B) or 35µM (C) CMLD-2 for 24h. The LDH activity was determined by measuring the absorbance at 490nm and 680nm.

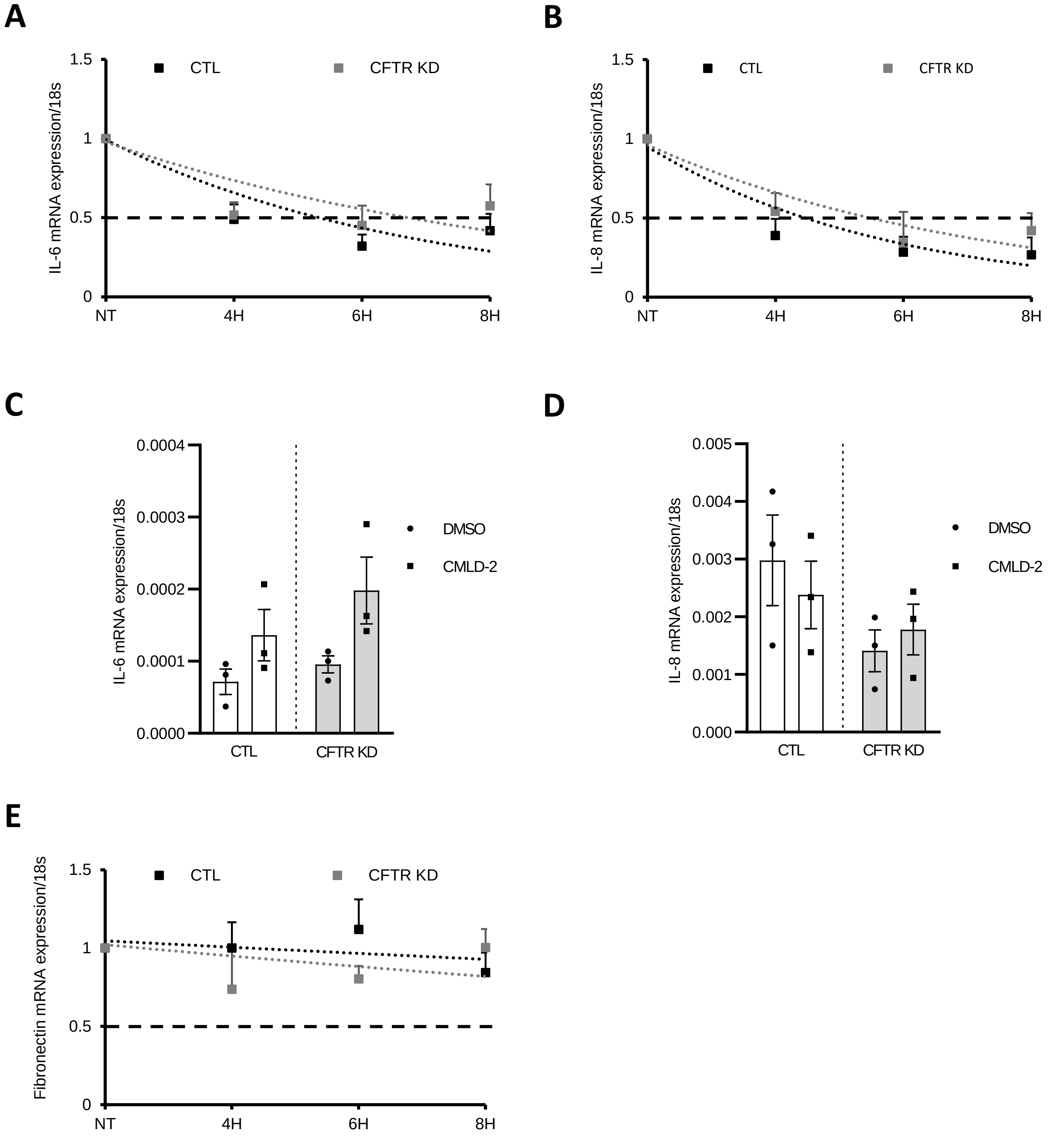

<u>Supplemental Figure 6.</u> **IL-6, IL-8 and fibronectin mRNA stability in CFTR KD cells *vs* CTL Calu-3 cells. A, B, E-** IL-6 (A), IL-8 (B) and fibronectin (E) mRNA decay was analyzed by RT-qPCR in CFTR KD *vs* CTL Calu-3 cells after inhibition of the *de novo* transcription by Actinomycin-D. n=3 in each group. Student's *t*-test, *p<0.05. **C, D-** Quantification of the relative mRNA expression of IL-6 (C) and IL-8 (D) by RT-qPCR in CFTR KD cells *vs* CTL Calu-3 cells treated with CMLD-2. 18s served as an internal control. n=3 in each group. Two-Way ANOVA, p>0.05.

A

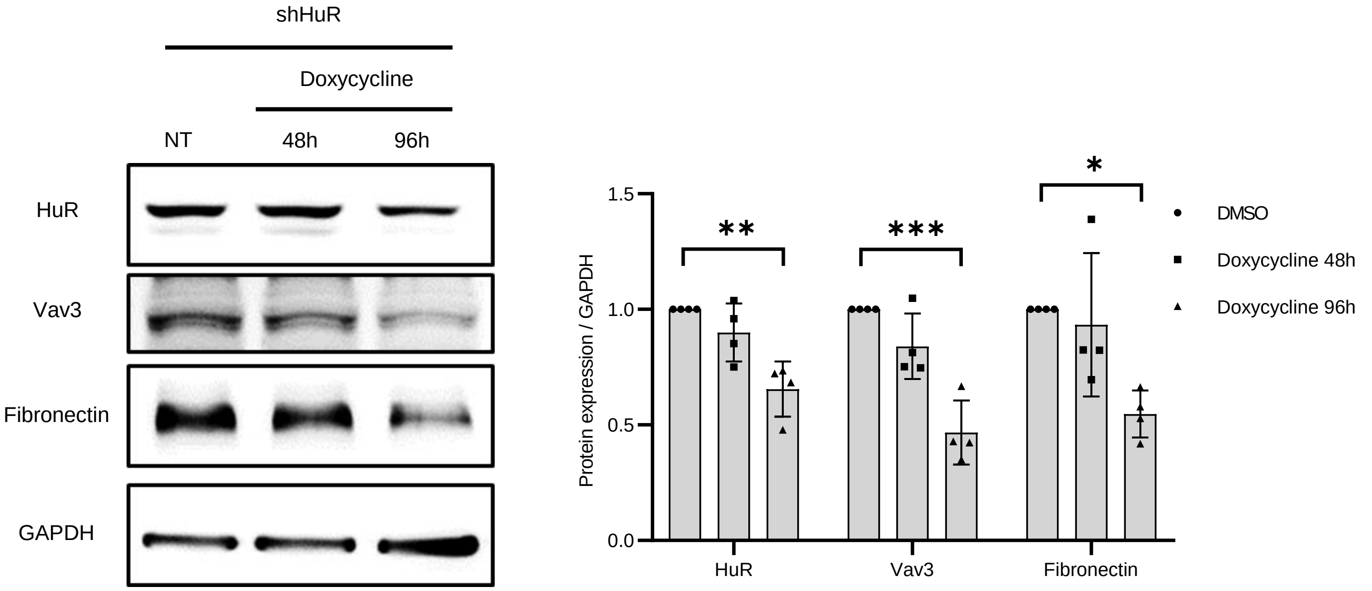

<u>Supplemental Figure 7.</u> Doxycycline-induced shHuR in CFTR KD Calu-3 cells decreased Vav3 and fibronectin expression. A- Representative Western blot showing HuR, Vav3 and fibronectin expression in CFTR KD after HuR silencing. GAPDH served as an internal control. The quantification is shown on the right panel. One-Way ANOVA, *p<0.05, **p<0.01, ***p<0.001.

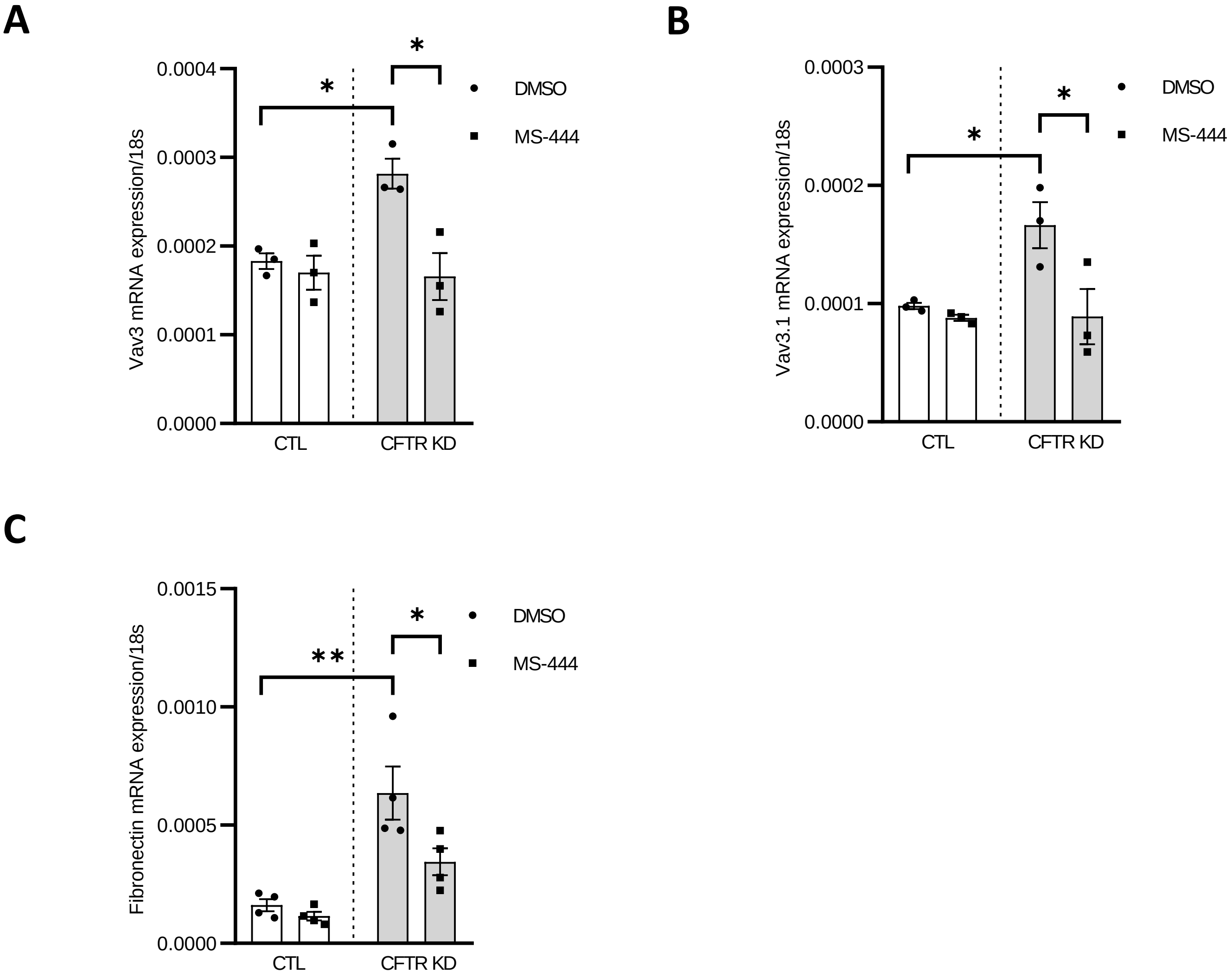

Supplemental Figure 8. MS-444 treatment decreased Vav3, Vav3.1 and fibronectin mRNA expression in CFTR KD. A, B, C- Quantification of the relative mRNA expression of Vav3 (A), Vav3.1 (B) and fibronectin (C) by RT-qPCR in CFTR KD cells *vs* CTL Calu-3 cells treated with MS-444. 18s served as an internal control. n=3 in each group for Vav3 and Vav3.1. n=4 in each group for fibronectin. Two-Way ANOVA, *p<0.05, **p<0.01.

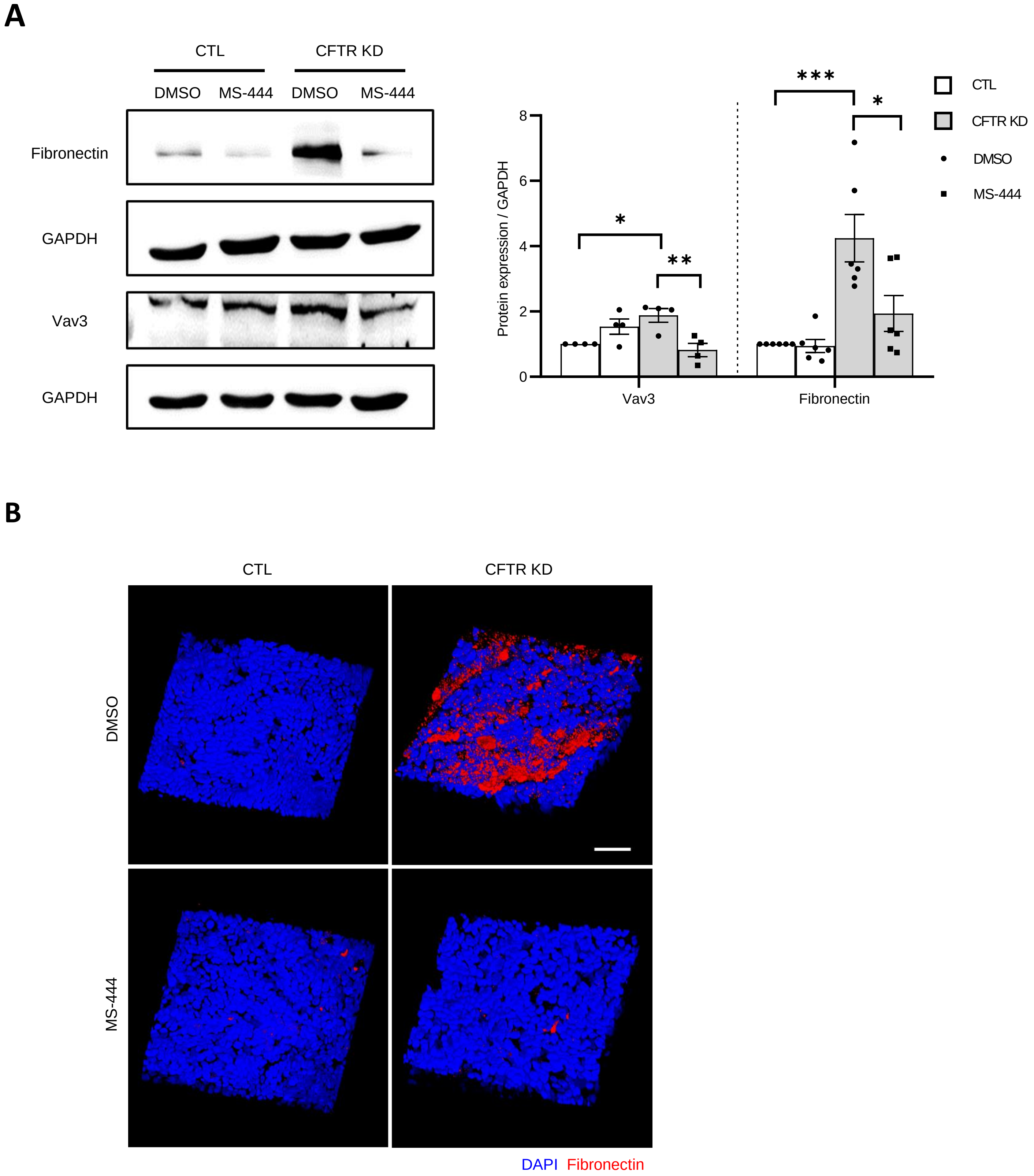

<u>Supplemental Figure 9.</u> MS-444 treatment prevented fibronectin apical deposition in CFTR KD cells. A- Representative Western blot showing Vav3 and fibronectin expression in CFTR KD cells *vs* CTL Calu-3 cells after MS-444 treatment. GAPDH served as an internal control. The quantifications are shown on the right panel. n=4 in each group for Vav3 and n=6 in each group for fibronectin. Two-Way ANOVA, *p<0.05, **p<0.01, ***p<0.001. **B-** Confocal microscopy analysis of fibronectin (red) in polarized CFTR KD cells *vs* CTL Calu-3 cells polarized at air-liquid interface after MS-444 treatment. Representative images from 3D reconstruction of Z-stack data are shown. Scale bar: 50µM.

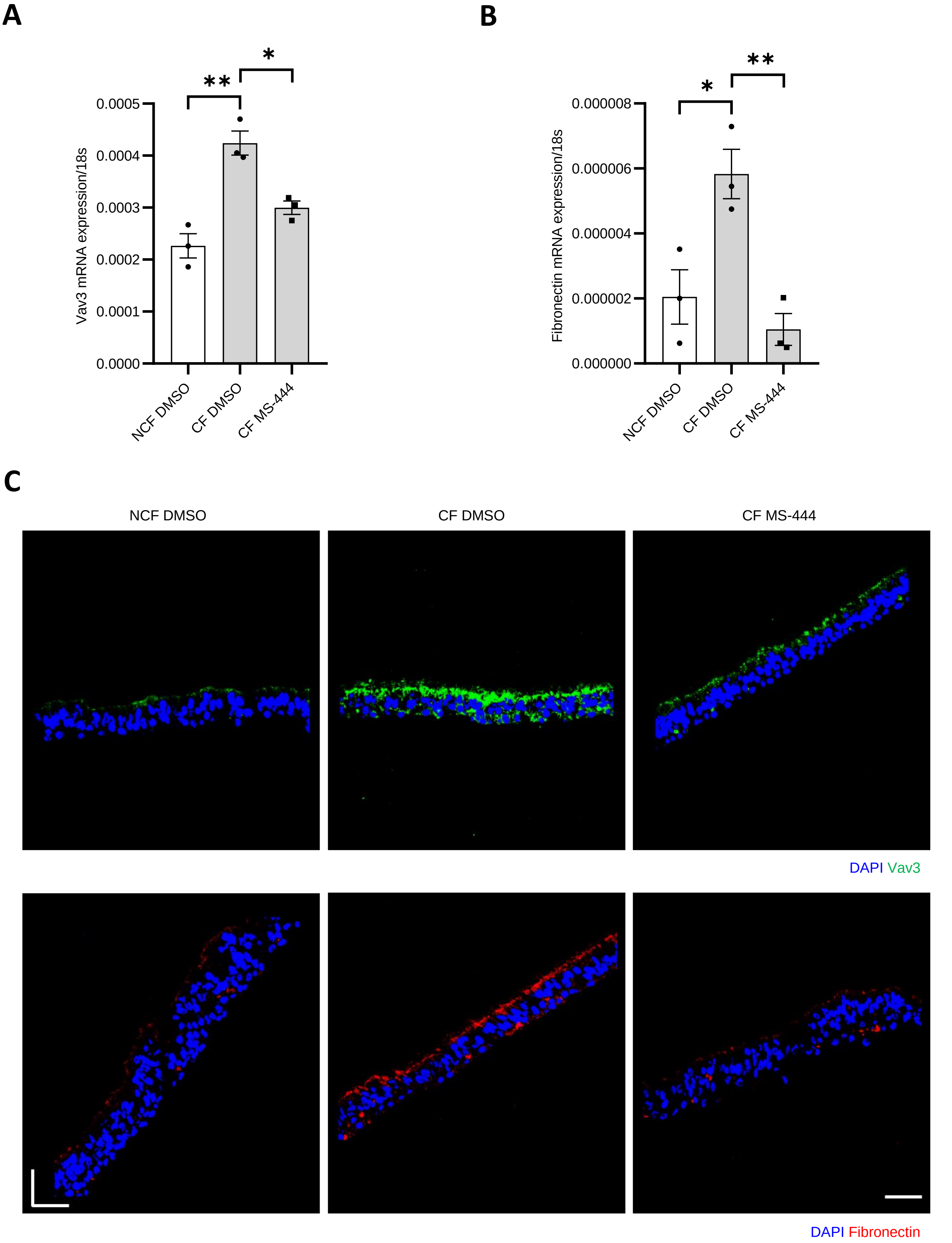

<u>Supplemental Figure 10.</u> HuR cytoplasmic translocation inhibition restored Vav3 and fibronectin normal expression in CF primary HAECs. A, B- Quantification of Vav3 (A) and fibronectin (B) mRNA expression by RT-qPCR in CF *vs* NCF fully differentiated primary HAECs following MS-444 treatment. 18s served as an internal control. 3 donors in NCF and 1 donor with 3 replicates in CF. Mann-Whitney test, *p<0.05, ** p<0.01. **C-** Representative confocal images of Vav3 (green) and fibronectin (red) immunostaining on cryosections of CF and NCF fully differentiated primary HAECs treated with MS-444. Nuclei are stained in blue. Scale bars: 40µM.

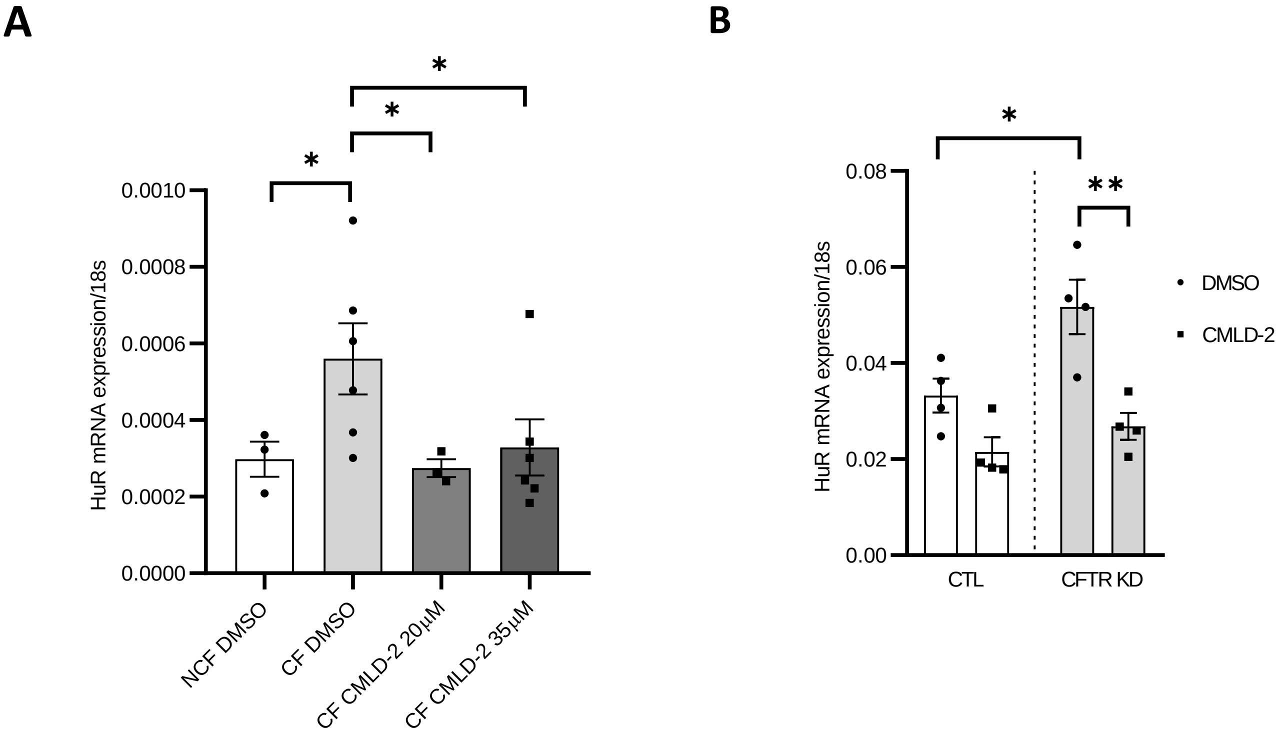

<u>Supplemental Figure 11.</u> HuR autoregulation in CF primary HAECs and CFTR KD Calu-3 cells. A- Quantification of HuR mRNA expression by RT-qPCR in CF *vs* NCF fully differentiated primary HAECs (A) following 72h CMLD-2 treatment. 18s served as an internal control. 3 donors in each group. Mann-Whitney test, *p<0.05. **B-** Quantification of HuR mRNA expression by RT-qPCR in CFTR KD cells *vs* CTL Calu-3 cells following 24h CMLD-2 treatment. 18s served as an internal control. n=4 in each group. Two-Way ANOVA, *p<0.05, **p<0.01.

	Age	Sex	Smoker	Pathology	Mutation	HAECs[1]	TEER[2] (Ω.cm^2)	Cilia beating frequency (Hz)
CF MD0567	39	Female	No	CF	Homozygous F508del	Bronchial	889+/-14	8.8+/-0.1
CF MD0607	21	Female	No	CF	Homozygous F508del	Bronchial	369+/-08	7.8+/-0.2
CF MD0526	16	Female	No	CF	Homozygous F508del	Bronchial	322+/-22	6.0+/-0.4
CF MD0220	29	Female	No	CF	Homozygous F508del	Bronchial	394+/-11	7.0+/-0.2
CF MD0485	25	-	No	CF	Homozygous F508del	Bronchial	232+/-16	7.9+/-0.1
CF MD0622	47	Female	No	CF	Homozygous F508del	Bronchial	369+/-11	8.1+/-0.1
CF MD0567	39	Female	No	CF	Homozygous F508del	Bronchial	528+/-16	7.5+/-0.1
NCF MD0805	17	Male	No	No Pathology reported	-	Bronchial	365+/-14	10.3+/-0.2
NCF MD0802	55	Female	No	No Pathology reported	-	Bronchial	297+/-07	9.0+/-0.4
NCF MD0670	15	Male	No	No Pathology reported	-	Bronchial	428+/-15	8.4+/-0.3
NCF MD0201	67	Female	-	No Pathology reported	-	Bronchial	656+/-09	7.0+/-0.2
NCF MD0537	61	Male	No	No Pathology reported	-	Bronchial	431+/-09	7.3+/-0.1
NCF MD0560	66	Male	-	No Pathology reported	-	Bronchial	331+/-12	7.8+/-0.1
NCF MD0835	35	Male	No	No Pathology reported	-	Bronchial	210+/-08	7.9+/-0.6
NCF MD0787	56	Female	No	No Pathology reported	-	Bronchial	295+/-17	11.2+/-0.5
NCF MD0801	27	Male	No	No Pathology reported	-	Bronchial	229+/-04	8.3+/-0.5

<u>Supplemental Table 1.</u> Clinicopathological characteristics of CF and NCF donors.

[1] Human Airway Epithelial Cells (HAECs). [2] TransEpithelial Electrical Resistance (TEER).

Gene	Forward (5’ to 3’)	Reverse (5’ to 3’)
HuR	AACTACGTGACCGCGAAGG	CGCCCAAACCGAGAGAACA
TTP	TCCACAACCCTAGCGAAGAC	GAGAAGGCAGAGGGTGACAG
Vav3	TCTGAAAGGAGATGCACACAGT	ACTGTGTGCATCTCCTTTCAGA
Vav3.1	CAAAATAACTTTACACTGACAATGCCAAT	ATTGGCATTGTCAGTGTAAAGTTATTTTG
Fibronectin	CACGGGAGCCTCGAAGAG	ACAACCGGGCTTGCTTTG
Il-6	AGAGGCACTGGCAGAAAACAA	AGGCAAGTCTCCTCATTGAAT
Il-8	ACTGAGAGTGATTGAGAGTGGAC	AACCCTCTGCACCCAGTTTTC
18s	GTAACCCGTTGAACCCCATT	CCATCCAATCGGTAGTAGCG

Supplemental Table 2. Primer sequences used for RT-qPCR experiments.

ANTIBODIES	SOURCE	IDENTIFIER
HuR	Cell Signaling	12582
TTP	Aviva systems biology	ARP38303-P050
Vav3	Sigma-Millipore	07-464
Total β1 integrin	Cell Signaling	4706
Active β1 integrin (Clone 9EG7)	BD Biosciences	553715
Pseudomonas aeruginosa	Abcam	AB68538
Flag peptide (IgG)	ABCD Antibodies	TA001
Fraction WB Cocktail	Abcam	AB140365
GAPDH	Millipore	MAB374
Fibronectin	Polyclonal antisera against human plasma fibronectin<br>(Gift from Dr B. Wehrle-Haller)	Clone 1801
Goat anti-Rabbit HRP	Sigma	A8275
Goat anti-Mouse HRP	Sigma	A5278
Alexa Fluor™ 647 Phalloidin	ThermoFisher	A22287
Alexa Fluor™ 488 goat anti-rabbit (H+L)	ThermoFisher	A11078
Alexa Fluor™ 568 goat anti-rabbit (H+L)	ThermoFisher	A11011
Alexa Fluor™ 647 goat anti-rabbit (H+L)	ThermoFisher	A21245
Alexa Fluor™ 568 goat anti-mouse (H+L)	ThermoFisher	A11031
Alexa Fluor™ 647 goat anti-mouse (H+L)	ThermoFisher	A21236

Supplemental Table 3. Antibodies used in this study.
